# Analysis of preoperative computed tomography radiomics and clinical factors for predicting postsurgical recurrence of papillary thyroid carcinoma

**DOI:** 10.1186/s40644-023-00629-9

**Published:** 2023-12-14

**Authors:** Haijun Xu, Wenli Wu, Yanfeng Zhao, Zhou Liu, Dan Bao, Lin Li, Meng Lin, Ya Zhang, Xinming Zhao, Dehong Luo

**Affiliations:** 1 Department of Radiology, National Cancer Center/National Clinical Research Center for Cancer/Cancer Hospital, Chinese Academy of Medical Sciences and Peking Union Medical College, Beijing, 100021 China; 2 Medical Imaging Center, Liaocheng Tumor Hospital, Liaocheng, 252000 China; 3 Department of Radiology, National Cancer Center/National Clinical Research Center for Cancer/Cancer Hospital & Shenzhen Hospital, Chinese Academy of Medical Sciences and Peking Union Medical College, Shenzhen, 518116 China

**Keywords:** Radiomics, Computed tomography, Papillary thyroid carcinoma, Recurrence, Nomogram

## Abstract

**Background:**

Postsurgical recurrence is of great concern for papillary thyroid carcinoma (PTC). We aim to investigate the value of computed tomography (CT)-based radiomics features and conventional clinical factors in predicting the recurrence of PTC.

**Methods:**

Two-hundred and eighty patients with PTC were retrospectively enrolled and divided into training and validation cohorts at a 6:4 ratio. Recurrence was defined as cytology/pathology-proven disease or morphological evidence of lesions on imaging examinations within 5 years after surgery. Radiomics features were extracted from manually segmented tumor on CT images and were then selected using four different feature selection methods sequentially. Multivariate logistic regression analysis was conducted to identify clinical features associated with recurrence. Radiomics, clinical, and combined models were constructed separately using logistic regression (LR), support vector machine (SVM), k-nearest neighbor (KNN), and neural network (NN), respectively. Receiver operating characteristic analysis was performed to evaluate the model performance in predicting recurrence. A nomogram was established based on all relevant features, with its reliability and reproducibility verified using calibration curves and decision curve analysis (DCA).

**Results:**

Eighty-nine patients with PTC experienced recurrence. A total of 1218 radiomics features were extracted from each segmentation. Five radiomics and six clinical features were related to recurrence. Among the 4 radiomics models, the LR-based and SVM-based radiomics models outperformed the NN-based radiomics model (P = 0.032 and 0.026, respectively). Among the 4 clinical models, only the difference between the area under the curve (AUC) of the LR-based and NN-based clinical model was statistically significant (P = 0.035). The combined models had higher AUCs than the corresponding radiomics and clinical models based on the same classifier, although most differences were not statistically significant. In the validation cohort, the combined models based on the LR, SVM, KNN, and NN classifiers had AUCs of 0.746, 0.754, 0.669, and 0.711, respectively. However, the AUCs of these combined models had no significant differences (all P > 0.05). Calibration curves and DCA indicated that the nomogram have potential clinical utility.

**Conclusions:**

The combined model may have potential for better prediction of PTC recurrence than radiomics and clinical models alone. Further testing with larger cohort may help reach statistical significance.

**Supplementary Information:**

The online version contains supplementary material available at 10.1186/s40644-023-00629-9.

## Background

Papillary thyroid carcinoma (PTC) is the most prevalent histological type of thyroid cancer, accounting for more than 80% of all thyroid malignancies. Unlike most other malignancies that focus on overall survival, postsurgical recurrence is of greater concern for PTC. Because of its indolent biological behavior, the mortality of patients with PTC is much lower, with an overall 10-year survival rate reaching ≥ 93% [1, 2]. However, the recurrence of PTC is relatively high, reported at 14–26% [1, 3–8], among whom, more than 30% may succumb to the disease [8]. One previous study with a total of 12 years of follow-up found that most patients developed recurrence within 5 years after surgery [9]. Furthermore, postsurgical recurrence exacerbates the psychological and economic burdens on patients. Thus, it is crucial to identify patients with PTC who are at high risk of recurrence so that individualized treatment can be tailored, including initial surgical planning, postoperative complementary therapy, intensiveness of postoperative supervision, and other management strategies.

It has been reported that tumor factors (e.g., aggressive histology, extrathyroidal extension (ETE), locoregional tissues invasion, and distant metastases) and lymph node (LN) factors (e.g., LN metastases, the size and number of involved LN) are important risk factors for PTC recurrence after surgery [7, 8, 10–14]. They were also included into the American Thyroid Association (ATA) risk stratification system declared in 2015 [15]. Computed tomography (CT), a routine clinical imaging modality, is capable of depicting detailed and objective anatomical information and, accordingly, can potentially provide many prognostic factors of patients with PTC [16, 17].

Moreover, large amounts of available data in CT images remain underutilized. Radiomics allows the conversion of digitally encrypted CT images into quantitatively mineable feature information on tumor morphology and pathophysiology, which may be related to clinical events in tumor management [18–20]. Radiomics based on CT has been demonstrated to be useful in predicting outcome for hepatocellular carcinoma, lung cancer, esophageal carcinoma, and colorectal cancer [21–27]. Remarkably, in those cancers, models that combined radiomics features with clinical risk factors achieved better performance than conventional approaches. Multiple studies have demonstrated that radiomics models and nomogram are useful for the prediction of PTC prognostic factors such as LN metastases and ETE [28–30]. And the usefulness of radiomics based on ultrasound for predicting PTC prognosis has been confirmed [31, 32].

However, to the best of our knowledge, no previous study has focused on CT-based radiomics to predict PTC recurrence. Hence, we aimed to investigate the value of CT-based radiomics features and conventional clinical factors in predicting the postsurgical recurrence of PTC.

## Methods

### Patients

**Fig. 1:**
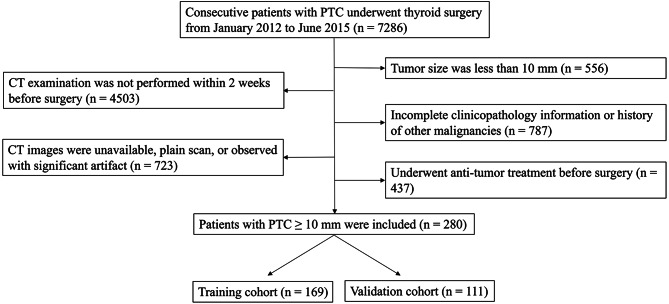
Flowchart of patient enrollment and allocation. CT: Computed tomography; PTC: Papillary thyroid carcinoma

This retrospective study was approved by our Institutional Review Board and the requirement for patient informed consent was waived. Between January 2012 and June 2015, 7286 consecutive patients underwent thyroid surgery at our institution. Our inclusion criteria were as follows: (a) pathologically confirmed papillary thyroid carcinoma with size ≥ 10 mm; (b) enhanced CT examination of the neck performed within 2 weeks before surgery; and (c) complete clinical and histopathology information. We excluded patients (a) for whom CT images were unavailable or degraded with significant artifacts; (b) who had a history of other malignancies, and (c) who underwent anti-tumor treatment before surgery. Finally, a total of 280 patients were recruited in our study. These patients were randomly divided into a training cohort (169 patients) and a validation cohort (111 patients) at a ratio of 6:4. The screening process is illustrated in Fig. 1. We utilized a combination of medical records review and telephone callbacks to follow up patients. Recurrence was defined as a cytology/pathology-proven disease or morphological evidence of lesions on imaging examinations detected within 5 years after surgery.

### Acquisition of clinicopathological characteristics

Clinicopathological characteristics were obtained from the patients’ medical records, and included age, sex, tumor size, number of metastatic LN, surgical options (subtotal or semi-total thyroidectomy, or total thyroidectomy), T stage, N stage, presence of bilaterality, multifocality, extrathyroidal extension, background concomitant thyroid diseases (e.g., nodular goiter and Hashimoto’s thyroiditis), history of ^131^I radiotherapy, and family history of PTC. The T and N stages were determined based on the seventh edition of the American Joint Committee on Cancer staging system.

### CT image evaluation

Preoperative enhanced CT was performed using three multi-slice spiral CT scanners (Discovery CT750 HD, Optima CT660, and LightSpeed VCT; GE Healthcare, Milwaukee, WI, USA). The imaging and post-processing protocols are detailed in Additional file 1. Two radiologists (Z.Y. and X.H.J., with 6 and 4 years of experience in head and neck imaging, respectively) independently reviewed the CT images from the picture archiving and communication system. Any differences were resolved by consensus through discussion and confirmed by L.L. (with 20 years of experience in head and neck imaging). All radiologists were blinded to the clinical outcomes. To be more precise and accurate, if multifocal lesions were present, we focused only on the largest lesion, while matching the corresponding tumor on the CT images to those examined by gross pathology based on location and size [13, 33]. The morphological CT image characteristics of the tumors were obtained, including shape (regular or irregular), margins (well-defined or ill-defined), presence of calcification, and CT-reported LN status (positive or negative). Positive LN status was defined by the presence of at least one of the following CT features: enhancement pattern (heterogeneous or rim), suspicious calcification, and cystic or necrotic changes, based on published criteria [34–37].

### Tumor segmentation and radiomics feature extraction

Reader 1 (W.W.L., with 15 years of experience in head and neck imaging) manually segmented the tumors on each consecutive transverse section of the contrast-enhanced CT images using ITK-SNAP (version 3.6.0, http://www.itksnap.org), which was then reviewed by a senior radiologist (L.L.). Interobserver consistency in tumor segmentation was assessed by calculating the Dice similarity coefficient (DSC). Readers 1 (W.W.L) and 2 (X.H.J.) independently segmented the tumors from the images of 30 randomly selected cases, before calculating the DSC between segmentations for absolute agreement from each case. A DSC index of more than 0.70 represented good consistency [38]. We carried out image pre-processing and feature extraction in FeAture Explorer (version 0.5.2), which incorporates the open-source package Pyradiomics [39]. To ensure a consistent intensity resolution across all tumor images, the voxel spacing was standardized using B-Spline interpolation, resampling the images to a voxel size of 1 × 1 × 1 mm^3^. Additionally, for gray-level discretization, a fixed bin width of 25 HU was chosen to reduce the noise and to normalize the intensity of the image.

### Feature reduction and selection

We extracted 1218 radiomics features from each three-dimensional segmentation. Least absolute shrinkage and selection operator (LASSO) regression was conducted to identify the most significant features related to recurrence. LASSO regression analysis is a penalized technique for feature selection of high-dimensional data to avoid overfitting [40]. Features with zero variance or high collinearity with other features (i.e., no variation or multicollinearity in feature values across patients) were removed from the analysis [41]. To reduce the redundancy of radiomics features, Pearson’s correlation analysis was conducted, and one of the paired features with a correlation coefficient > 0.6 was eliminated. Multivariate logistic regression analysis was performed using the backward selection method to identify clinical features associated with recurrence. Akaike information was used as the stopping criterion.

### Model construction and validation

Radiomics, clinical, and combined models were constructed separately using the following four classifiers for predicting PTC recurrence: logistic regression (LR), support vector machine (SVM), k-nearest neighbor (KNN), and neural network (NN). The radiomics model was established on selected radiomics features, the clinical model was established on independent clinical features, and the combined model was established on both radiomics and clinical features. Receiver operating characteristic (ROC) curves were plotted, and the area under the curve (AUC) was calculated to evaluate the predictive performance of these models in the training and validation cohorts.

### Model interpretation

**Fig. 2:**
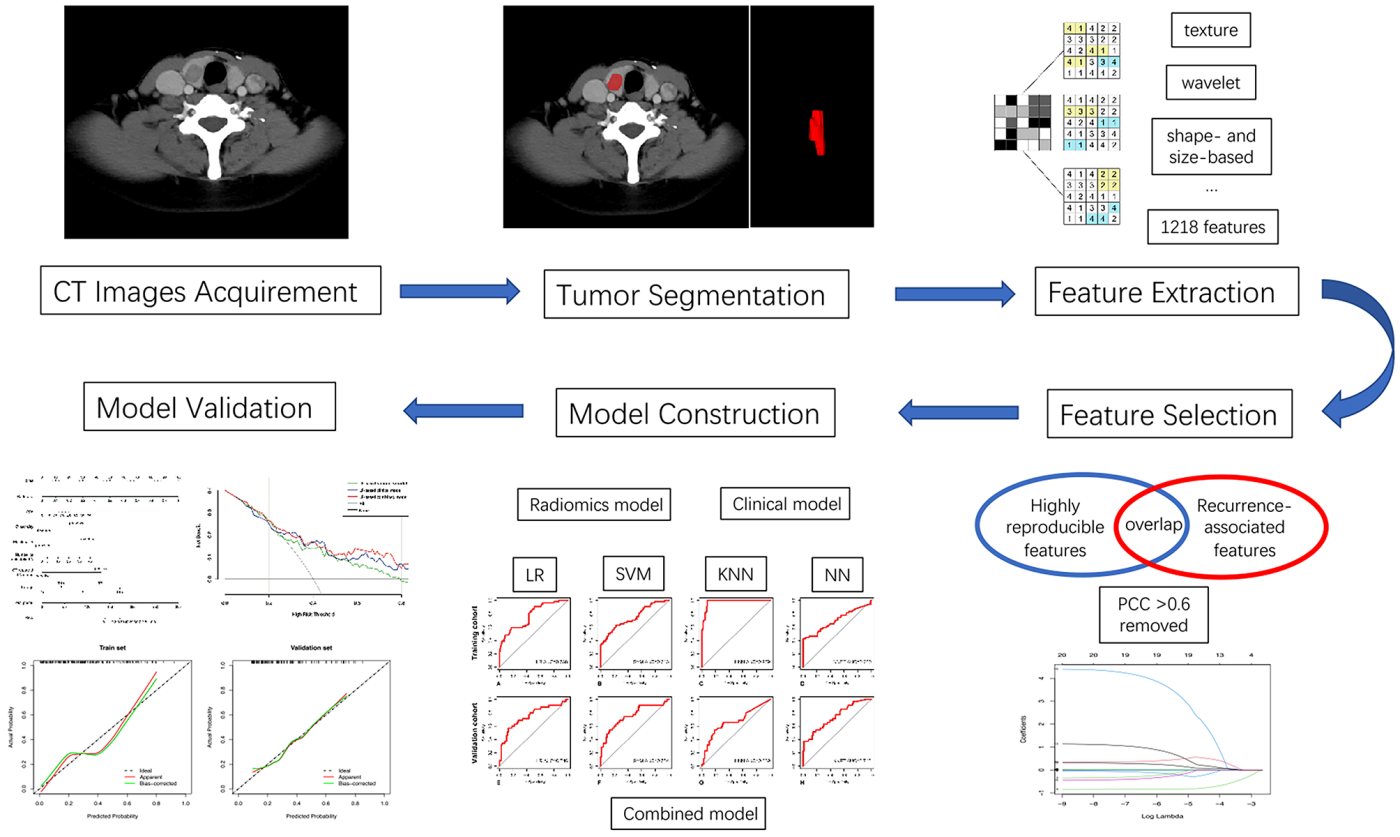
Workflow scheme of the radiomics analysis. KNN: K-nearest neighbor; LR: Logistic regression; NN: Neural network; PCC: Pearson’s correlation coefficient; SVM: Support vector machine

A respective radiomics signature (named “Rad-score”) was constructed by combining selected radiomics features. We then constructed a nomogram in R using the “rms” package, which involved integrating the Rad-score and the selected clinical risk factors. Calibration curves of the nomogram were obtained by plotting the predicted probability and actual observed proportion of recurrence using bootstraps with 1000 resamples. A good degree of calibration was achieved when the curve approximated the diagonal line, indicating high accuracy of the nomogram [42]. Decision curve analysis (DCA) was implemented in R using the “rmda” package, which involved the quantification of the clinical net benefits at a variety of risk threshold probabilities. The highest curve at a given threshold probability indicates the best prediction model with potential clinical utility in predicting PTC recurrence [43]. Figure 2 shows the workflow of the radiomics analysis.

### Statistical analysis

The Shapiro–Wilk test was used to determine whether the distribution of continuous variables was normal. Comparisons of the clinical features were performed using the Student’s t-test or Mann–Whitney U test for continuous variables and the chi-square test or Fisher’s exact test for categorical variables, as applicable. The DeLong test was used to compare the AUC of different models. The accuracy, specificity, and sensitivity of the models were calculated for each model. All statistical analyses were performed using R (version 4.0.2) and SPSS (version 26.0) software. All levels of statistical significance were two-sided, and P-values < 0.05 were considered a statistically significant difference.

## Results

### Characteristics of the study cohort

A total of 280 patients with pathologically confirmed PTC, including 186 women (40.40 ± 13.71 years; range: 19–77 years) and 94 men (41.40 ± 13.46 years; range: 21–70 years), were enrolled in this study. One hundred and seventy-nine patients were regularly followed up at our hospital, and the other 101 patients were called for the prognosis information. The training and validation cohorts had similar clinicopathological-radiologic characteristics of the patients, with no significant differences, which are summarized in Table 1. The clinicopathologic and morphological CT features of patients with and without disease recurrence were compared in Table 2, which showed significant differences in age, sex, tumor size, number of metastatic LN, T stage, N stage, bilaterality, history of ^131^I radiotherapy, and CT-reported LN status. For patients with and without recurrence, the follow-up period was 6 to 59 months (median 14.5 months) and 60 to 112 months (median 78 months), respectively. Consequently, the follow-up durations for the study cohort ranged from 6 to 112 months, with 68 months as the median. During follow-up, recurrence was detected in 89 patients within 5 years after surgery.

**Table 1:** Baseline characteristics of patients in the training and validation cohorts

Characteristic	Training cohort (n = 169)	Validation cohort (n = 111)	P-value
Recurrence			0.941
Absence	115 (68.0)	76 (68.5)	
Presence	54 (32.0)	35 (31.5)	
Clinicopathologic features			
Age (years)^a^	40.25 ± 13.71	41.47 ± 13.49	0.466
Sex			0.479
Male	54 (32.0)	40 (36.0)	
female	115 (68.0)	71 (64.0)	
Tumor size (mm)^a^	20.36 ± 10.20	22.31 ± 11.85	0.156
Bilaterality			0.572
Absence	85 (50.3)	52 (46.8)	
Presence	84 (49.7)	59 (53.2)	
Multifocality			0.383
Absence	65 (38.5)	37 (33.3)	
Presence	104 (61.5)	74 (66.7)	
Extrathyroidal extension			0.161
Absence	13 (7.7)	4 (3.6)	
Presence	156 (92.3)	107 (96.4)	
Number of metastatic LN^b^	11 (5–19)	10 (4–19)	0.874
Concomitant thyroid disease			0.107
Neither	43 (25.4)	30 (27.0)	
Nodular goiter	80 (47.3)	62 (55.9)	
Hashimoto’s thyroiditis	41 (24.3)	14 (12.6)	
Both	5 (3.0)	5 (4.5)	
Surgical option			0.302
Total thyroidectomy	140 (82.8)	97 (87.4)	
Subtotal or semi-total thyroidectomy	29 (17.2)	14 (12.6)	
T stage			0.697
T1a	3 (1.8)	0 (0)	
T1b	6 (3.6)	3 (2.7)	
T2	2 (1.2)	1 (0.9)	
T3	137 (81.1)	96 (86.5)	
T4a	21 (12.4)	11 (9.9)	
N stage			0.897
N0	17 (10.1)	10 (9.0)	
N1a	23 (13.6)	17 (15.3)	
N1b	129 (76.3)	84 (75.7)	
^131^I radiotherapy			0.359
Not received	88 (52.1)	64 (57.7)	
Received	81 (47.9)	47 (42.3)	
Family history			0.385
Absence	159 (94.1)	107 (96.4)	
Presence	10 (5.9)	4 (3.6)	
CT features			
Shape			0.594
Regular	30 (17.8)	17 (15.3)	
Irregular	139 (82.2)	94 (84.7)	
Margin			0.588
Well-defined	24 (14.2)	19 (17.1)	
Ill-defined	145 (85.8)	92 (82.9)	
Calcification			0.172
Absence	81 (47.9)	44 (39.6)	
Presence	88 (52.1)	67 (60.4)	
CT-reported LN status			0.291
Negative	35 (20.7)	29 (26.1)	
Positive	134 (79.3)	82 (73.9)	

Unless otherwise indicated, data are the number of patients and data in parentheses are percentages

^a^ Data are means ± standard deviation

^b^ Data are medians, with interquartile range in parentheses

CT: computed tomography; LN: lymph nodes

**Table 2:** Baseline characteristics of patients with and without disease recurrence

Characteristic	All patients (n = 280)	No recurrence (n = 191)	Recurrence (n = 89)	P-value
Clinicopathologic features				
Age (years)^a^	40.74 ± 13.61	39.07 ± 11.91	44.31 ± 16.19	0.003^*^
Sex				0.019^*^
Male	94 (33.6)	55 (28.8)	39 (43.8)	
female	186 (66.4)	136 (71.2)	50 (56.2)	
Tumor size (mm)^a^	24.58 ± 14.61	22.74 ± 11.76	28.52 ± 18.84	0.002^*^
Bilaterality				0.005^*^
Absence	137 (48.9)	105 (55.0)	32 (36.0)	
Presence	143 (51.1)	86 (45.0)	57 (64.0)	
Multifocality				0.114
Absence	102 (36.4)	76 (39.8)	26 (29.2)	
Presence	178 (63.6)	115 (60.2)	63 (70.8)	
Extrathyroidal extension				0.119
Absence	17 (6.1)	15 (7.9)	2 (2.2)	
Presence	263 (93.9)	176 (92.1)	87 (97.8)	
Number of metastatic LN^b^	11 (4–19)	10 (3–17)	15 (8–24)	< 0.001^*^
Concomitant thyroid disease				0.067
Neither	73 (26.1)	44 (23.0)	29 (32.6)	
Nodular goiter	142 (50.7)	96 (50.3)	46 (51.7)	
Hashimoto’s thyroiditis	55 (19.6)	45 (23.6)	10 (11.2)	
Both	10 (3.6)	6 (3.1)	4 (4.5)	
Surgical option				0.678
Total thyroidectomy	237 (84.6)	160 (83.8)	77 (86.5)	
Subtotal or semi-total thyroidectomy	43 (15.4)	31 (16.2)	12 (13.5)	
T stage				0.001^*^
T1a	3 (1.1)	3 (1.6)	0 (0.0)	
T1b	9 (3.2)	7 (3.7)	2 (2.2)	
T2	3 (1.1)	3 (1.6)	0 (0.0)	
T3	233 (83.2)	166 (86.9)	67 (75.3)	
T4a	32 (11.4)	12 (6.3)	20 (22.5)	
N stage				0.044^*^
N0	27 (9.6)	24 (12.6)	3 (3.4)	
N1a	40 (14.3)	28 (14.7)	12 (13.5)	
N1b	213 (76.1)	139 (72.8)	74 (83.1)	
^131^I radiotherapy				0.044^*^
Not received	152 (54.3)	112 (58.6)	40 (44.9)	
Received	128 (45.7)	79 (41.4)	49 (55.1)	
Family history				0.576
Absence	266 (95.0)	180 (94.2)	86 (96.6)	
Presence	14 (5.0)	11 (5.8)	3 (3.4)	
CT features				
Shape				0.842
Regular	12 (4.3)	9 (4.7)	3 (3.4)	
Irregular	268 (95.7)	182 (95.3)	86 (96.6)	
Margin				0.510
Well-defined	11 (3.9)	9 (4.7)	2 (2.2)	
Ill-defined	269 (96.1)	182 (95.3)	87 (97.8)	
Calcification				0.275
Absence	125 (44.6)	90 (47.1)	35 (39.3)	
Presence	155 (55.4)	101 (52.9)	54 (60.7)	
CT-reported LN status				0.009^*^
Negative	31 (11.1)	28 (14.7)	3 (3.4)	
Positive	249 (88.9)	163 (85.3)	86 (96.6)	

Unless otherwise indicated, data are the number of patients and data in parentheses are percentages

^a^ Data are means ± standard deviation

^b^ Data are medians, with interquartile range in parentheses

^*^Statistically significant difference

CT: computed tomography; LN: lymph nodes

### Feature extraction and selection

Two readers showed good consistency in manual segmentation with a DSC of 0.83 ± 0.04 (range: 0.76–0.90). A total of 1218 radiomics features were initially extracted, which are listed in Additional file 2. One-hundred and seventy features related to recurrence were selected after LASSO regression. No features had a near-zero variance, and one feature was removed from the dataset because of its high collinearity with other features. After Pearson’s correlation analysis, the following five radiomics features associated with recurrence were selected: original-shape-sphericity, log-sigma-2-0-mm-3D-GLCM (Grey-Level co-occurrence matrix)-informational measure of correlation 2, wavelet-HLL-firstorder-mean, log-sigma-3-0-mm-3D-firstorder-90 percentile, and wavelet-LLL-firstorder-skewness. A flowchart of the radiomics feature selection process is shown in Additional file 3. After multivariate logistic regression analysis using backward selection, six clinical features, including age, number of metastatic LN, CT-reported LN status, T stage, presence of bilaterality, and multifocality, were identified to be related to recurrence (Table 3). The first three radiomics features and the presence of multifocality were negatively correlated with PTC recurrence, whereas the other features were positively correlated.

**Table 3:** Clinical features associated with recurrence identified by multivariate logistic regression analysis using backward selection

Clinical features	β	Wald	SE	P-value	OR (95% CI)
Age	0.026	3.373	0.014	0.07	1.027 (0.999–1.057)
Bilaterality	1.168	2.049	0.816	0.15	3.216 (0.770-22.095)
Multifocality	-1.399	2.874	0.825	0.09	0.247 (0.035–1.050)
Number of metastatic LN	0.044	4.119	0.022	0.04	1.045 (1.002–1.091)
CT-reported LN status	1.816	2.789	1.088	0.09	6.149 (1.060-117.379)
T stage	0.911	4.047	0.453	0.04	2.487 (1.109–6.495)
Intercept	-7.598	13.445	2.072	< 0.001	0.001 (0-0.019)

LN: Lymph node; OR: Odds ratio; SE: Standard error

### Model development and validation

**Fig. 3:**
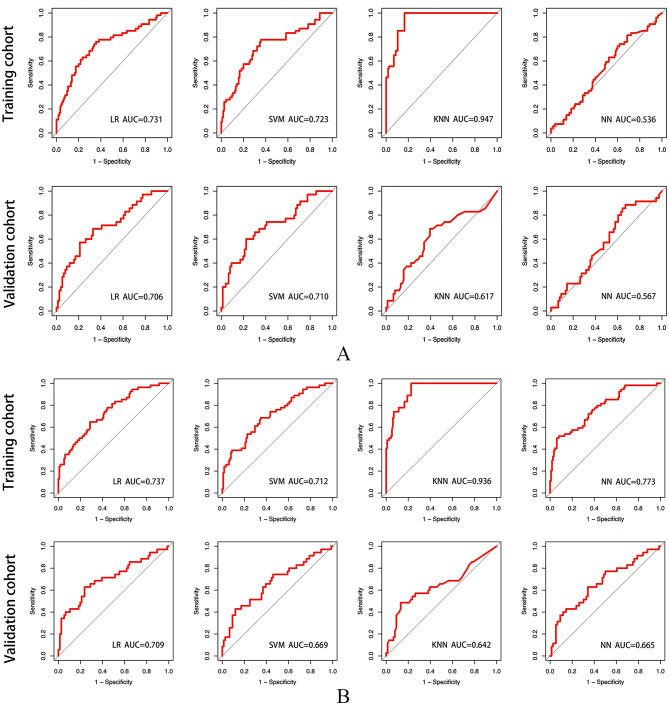
Receiver operating characteristic (ROC) curves for the radiomics models (A) and clinical models (B). AUC: Area under the receiver operating characteristic curve; KNN: K-nearest neighbor; LR: Logistic regression; NN: Neural network; SVM: Support vector machine

**Fig. 4:**
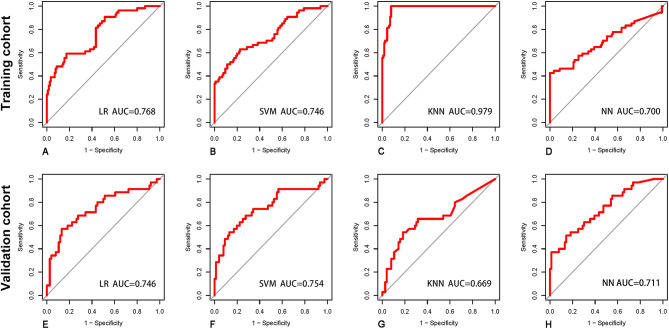
Receiver operating characteristic (ROC) curves for the combined models. AUC: Area under the receiver operating characteristic curve; KNN: K-nearest neighbor; LR: Logistic regression; NN: Neural network; SVM: Support vector machine

**Fig. 5:**
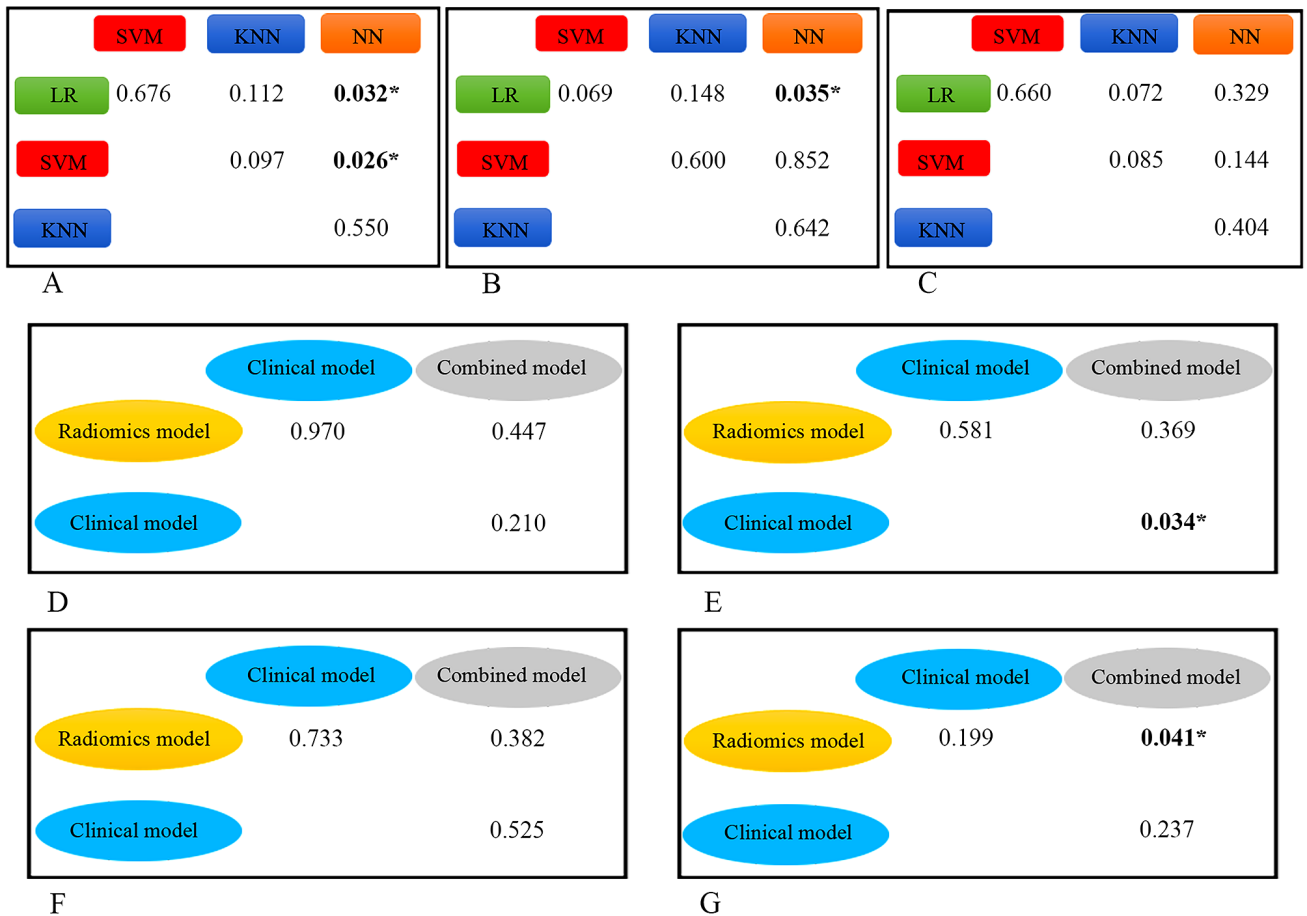
The P-values calculated by comparing the AUC between models using the Delong test. (A: radiomics model, B: clinical model, C: combined model) The P-value was calculated by comparing the AUC of the model with that of the same type of model based on another classifier. (D: LR classifier, E: SVM classifier, F: KNN classifier, G: NN classifier) The P-value was calculated by comparing the AUC of the model with that of another model based on the same classifier. *Statistically significant difference. AUC: Area under the receiver operating characteristic curve; KNN: K-nearest neighbor; LR: Logistic regression; NN: Neural network; SVM: Support vector machine

The ROC curves for the radiomics, clinical, and combined models in the training and validation cohorts are shown in Figs. 3 and 4. The predictive performances of the models in the validation cohort are detailed in Table 4. Figure 5 shows the P-value calculated using Delong test to compare the AUC values of the models. Among the 4 radiomics models, the LR-based and SVM-based radiomics models outperformed the NN-based radiomics model (P = 0.032 and 0.026, respectively). Among the 4 clinical models, only the difference between the AUC of the LR-based and NN-based clinical model was statistically significant (P = 0.035). The combined models had higher AUC values than the corresponding radiomics and clinical models based on the same classifier, although most differences were not statistically significant. However, the SVM-based combined model significantly outperformed the clinical model, and the NN-based combined model had significant improvement than the radiomics model (P = 0.034 and 0.041, respectively). This finding indicates that the combination of radiomics and clinical features have potential improvement in predicting recurrence of PTC. In the validation cohort, the AUCs of the combined models based on the LR, SVM, KNN, and NN classifiers were 0.746 (95% [confidence interval (CI)]: 0.640–0.852), 0.754 (95% CI: 0.649–0.859), 0.669 (95% CI: 0.552–0.785), and 0.711 (95% CI: 0.607–0.816), respectively. The accuracies were 0.739, 0.766, 0.730 and 0.766, respectively. However, the AUCs of these combined models had no significant differences (all P > 0.05).

**Table 4:** Performance of the models for predicting PTC recurrence in the validation cohort

Model	Classifier	AUC	95% CI		ACC	SPE	SEN
			Low	High			
Radiomics models	LR	0.706	0.601	0.812	0.739	0.908	0.371
	SVM	0.710	0.604	0.816	0.721	0.961	0.200
	KNN	0.617	0.499	0.735	0.676	0.842	0.314
	NN	0.567	0.454	0.679	0.685	1.000	0
Clinical models	LR	0.709	0.597	0.821	0.748	0.895	0.429
	SVM	0.669	0.556	0.782	0.694	0.934	0.171
	KNN	0.642	0.522	0.763	0.739	0.855	0.486
	NN	0.665	0.552	0.778	0.730	0.895	0.371
Combined models	LR	0.746	0.640	0.852	0.739	0.895	0.400
	SVM	0.754	0.649	0.859	0.766	0.921	0.429
	KNN	0.669	0.552	0.785	0.730	0.842	0.486
	NN	0.711	0.607	0.816	0.766	0.947	0.371

ACC: Accuracy; AUC: Area under the receiver operating characteristic curve; CI: Confidence interval; KNN: K-nearest neighbor; LR: Logistic regression; NN: Neural network; SEN: Sensitivity; SPE: Specificity; SVM: Support vector machine

### Model interpretation with the nomogram and DCA

The formula for calculating the Rad-score is as follows:

log (Rad-score) = 11.504 + -12.463 × original-shape-sphericity + -4.323 × log-sigma-2-0-mm-3D-GLCM-informational measure of correlation 2 + -0.049 × wavelet-HLL-firstorder-mean + 0.014 × log-sigma-3-0-mm-3D-firstorder-90 percentile + 0.001 × wavelet-LLL-firstorder-skewness.

**Fig. 6:**
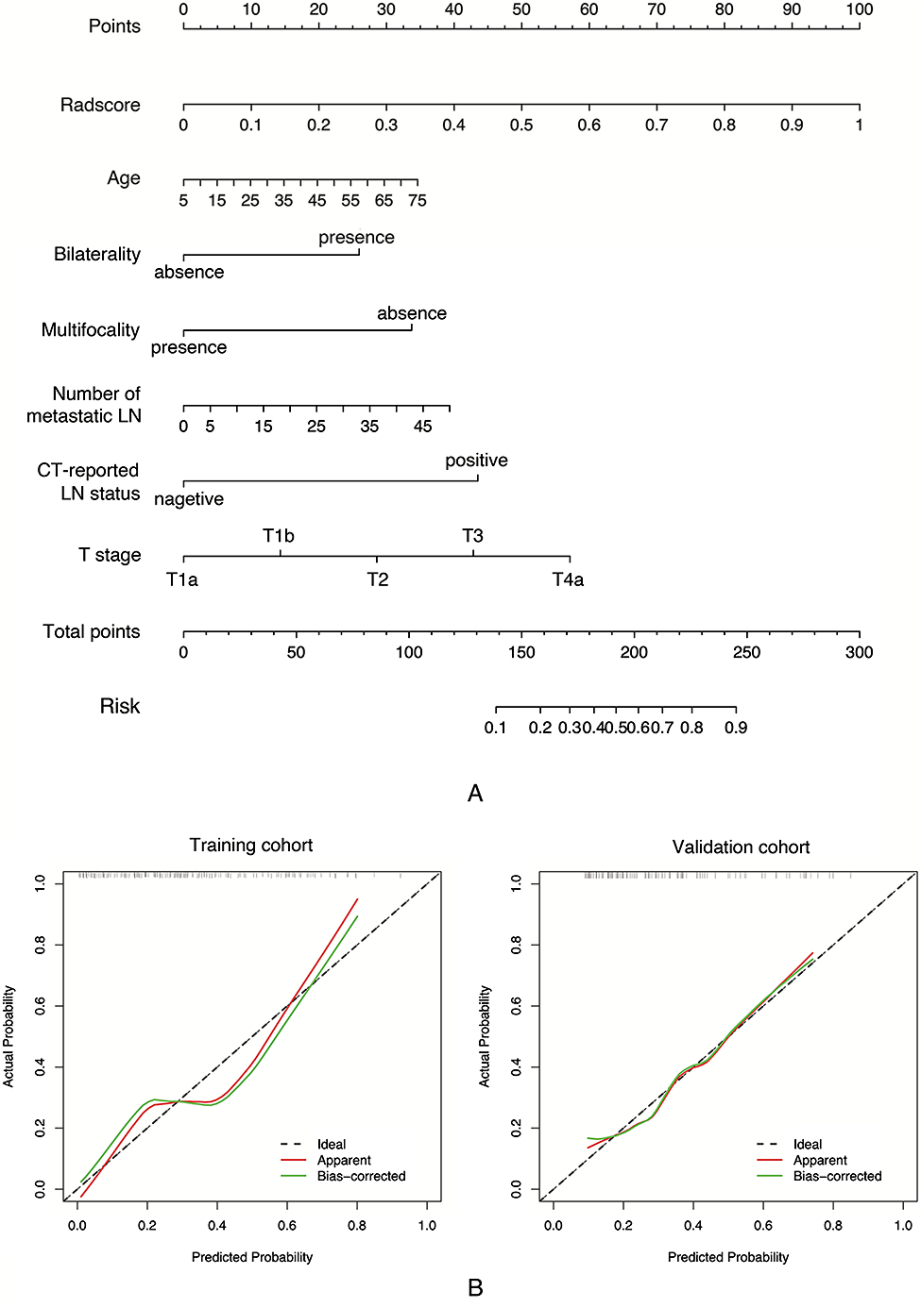
Nomogram established to provide individualized risk estimates (A), which was verified by calibration curves (B). Calibration curves describe the agreement between the probability of recurrence predicted by the nomogram and the actual positive proportion of recurrence. The red and green solid lines represent the apparent and bias-corrected predictive performances of the nomogram, respectively, whereas the diagonal line represents the ideal performance

**Fig. 7:**
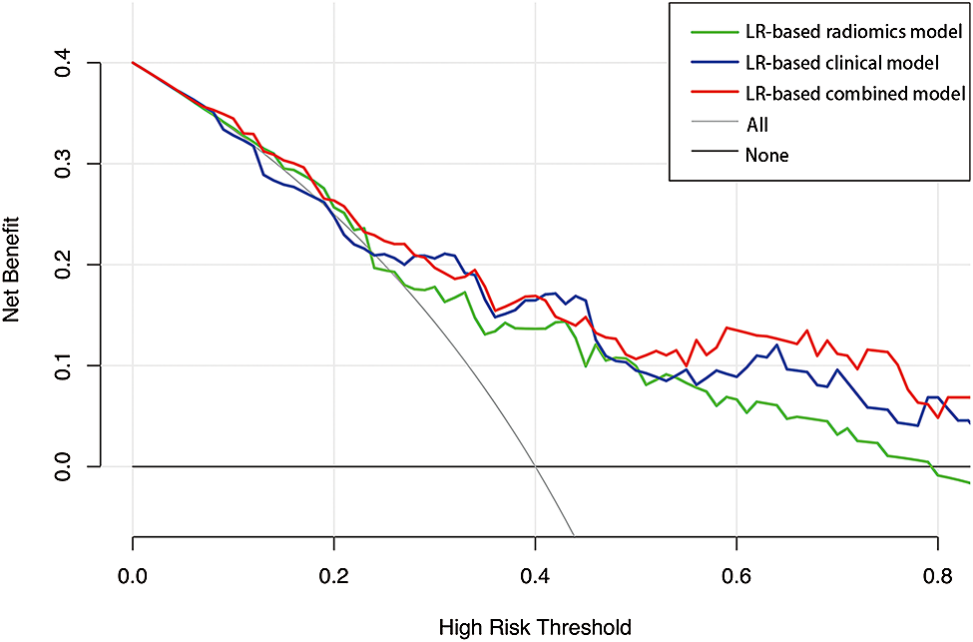
Decision curve analysis of models for predicting PTC recurrence in the validation cohort. The x-axis represents the threshold probability. The y-axis represents the net benefit calculated by summing the benefits (true-positive results) and subtracting the harms (false-positive results). The LR-based combined model provided the highest net benefit compared to simple strategies (tracking all patients or no patients) and the other two models when the patients’ threshold probability was within a reasonable range. LR: Logistic regression; PTC: Papillary thyroid carcinoma

A nomogram was constructed by combining the Rad-score with six clinical features using multivariate logistic regression to provide individualized risk estimates (Fig. 6A). The calibration curves of the nomogram approximated the diagonal line in Fig. 6B, especially in the validation cohort, indicating high accuracy for recurrence prediction. According to the DCA results (Fig. 7), the LR-based combined model provided the highest net benefit in comparison to the other two models and simple strategies (tracking all patients or none) when the patients’ threshold probability was within a reasonable range.

## Discussion

In this study, we identified 5 radiomics features and 6 clinical risk factors that were associated with postsurgical recurrence of PTC. Radiomics, clinical, and combined models were constructed using four classifiers (LR, SVM, KNN, and NN) to predict PTC recurrence after the initial surgery. In the validation cohort, all models had favorable accuracy (0.676–0.766) and specificity (0.842-1) but relatively low sensitivity (0-0.486). The combined model integrating radiomics and clinical features exhibited highest AUC in the validation cohort. The nomogram incorporating all features enable us to obtain patient’s risk of postoperative recurrence in a short period of time after surgery, which will provide certain help to evaluate treatment schedules and follow-up protocols.

Only 280 of 7286 assessed patients were included in the final study. According to the ATA guidelines for the management of patients with differentiated thyroid cancer [15], the majority of patients with PTC did not undergo CT examination before surgery, which was the main exclusion factor of our investigation. Together with the influence of other factors (including incomplete clinical data, image artifacts, etc.), most patients were excluded. However, patients with larger and more advanced tumors tend to receive CT examination to detect the involvement extent. Our findings are valuable for predicting postoperative recurrence probability of these patients with PTC.

In our study, one shape-based feature (i.e., original-shape-sphericity), one texture feature (i.e., log-sigma-2-0-mm-3D-GLCM-informational measure of correlation 2), and three first-order features (i.e., wavelet-HLL-firstorder-mean, log-sigma-3-0-mm-3D-firstorder-90 percentile, and wavelet-LLL-firstorder-skewness) were selected as relevant predictors of PTC recurrence, and the first three were negatively correlated with the outcomes. Sphericity, which describes and quantifies the spherical shape of a tumor, has been reported as a stable radiomics shape feature that is rarely influenced by slice thickness, volume, or resampling [44]. A less spherical shape corresponds to an irregular tumor morphology, which is usually associated with a more aggressive nature of PTC, such as a larger tumor size and gross extrathyroidal invasion. Thus, consistently, a lower tumor sphericity was associated with a higher risk of recurrence in our study. In addition, GLCM features can quantify textural information and can be used to identify intra-tumor heterogeneity for a variety of cancer types [23, 45–47]. The information measure of correlation is a GLCM-based feature associated with the joint probability of the occurrence of pixel pair entropy. A lower value of this feature indicates a higher heterogeneity in the distribution of intensities of PTC. In our study, PTC with high risk of recurrence after surgery had higher heterogeneity in the intra-lesion texture, which needs further confirmation. Besides, quantifying gray-level frequency distribution, three first-order features selected were associated with the risk of postsurgical recurrence. Two of these features were extracted after wavelet transformation to measure more complex tumor heterogeneity parameters by reflecting the image properties at different scales and orientations [24, 25]. The value of these five radiomics features in predicting recurrence in patients with PTC needs confirmation in the future.

In this study, age, number of metastatic LN, and T stage were identified as high-risk clinicopathological factors for PTC recurrence, which is concordant with the results of previous findings [6, 7, 10, 11]. Another study has reported that the number of metastatic cervical LNs and the ratio of metastatic-to-total dissected cervical LNs are the main risk factors for PTC recurrence among patients younger than 55 years of age, while the size of the thyroid lesions (T stage) was recognized as a predictor in patients older than 55 years of age [14]. In addition, bilaterality and multifocality were associated with PTC recurrence, however, the roles of which in predicting recurrence of PTC are controversial [48–51]. Notably, multifocality was negatively correlated with PTC recurrence risk in this study. One possible reason is that most patients with multifocal tumors underwent total thyroidectomy (174/178) and therefore had a lower risk of locoregional recurrence compared to patients who underwent thyroid lobectomy because of more thorough surgical involvement. Furthermore, the proportion of multifocal lesions (63.6%) was much higher than that of previous studies (less than 40%) [52–55], and there may be a selection bias that affects the final accuracy of the results, which needs to be further confirmed in future studies. CT-reported LN status, a preoperative prognostic risk factor, may prompt the surgeons to determine the extent of surgical resection and LN dissection. It was obtained from medical images before surgery and was also incorporated into the models, suggesting the morphology of lymph nodes on CT images may be useful in predicting PTC recurrence after surgery.

Additionally, one previous study reported a positive relationship between PTC recurrence and ETE predicted by radiomics-based nomogram [30]. In our study, ETE was not relevant to the risk of recurrence, probably because of the inclusion of microscopic ETE, which has been reported to have no effect on PTC recurrence in several studies [55–57]. Kim et al. concluded that microscopic ETE was not associated with recurrence (p = 0.081), whereas macroscopic ETE was an independent risk factor for poor prognosis with a 13-fold increased relative risk of recurrence [57]. However, some studies argued that patients with PTC who have microscopic ETE are at an increased risk of recurrence compared to patients without ETE [58, 59]. Therefore, the role of ETE in predicting the recurrence of PTC requires further investigation through specialized design.

Our study has some limitations that warrant discussion. First, we used CT images with a 5-mm slice thickness because those with a thinner thickness were unavailable for most of our cohort, which might have influenced the detectability of the tumor and the accuracy of segmentation. However, all tumors were visible on at least two consecutive images because we only segmented lesions larger than 10 mm in diameter. And the manual segmentation had good accuracy and reproducibility because of a confirmation by a senior radiologist and a good interobserver consistency (DSC = 0.83). Second, for those patients with multiple lesions, we focused only on the single largest lesion, because it is challenging to delineate all tumors, which may not accurately reflect the tumor burden. Third, the recurrence rate (31.8%) is higher than that reported in some previous studies [1, 3–8]. This may be partly attributed to the exclusion of microcarcinomas in our study, which exhibit a relatively good prognosis. Finally, after four different feature selection methods, out of a total of 1218 radiomics features extracted from each segmentation, only 5 were found to be related to recurrence of PTC. Independent external validation datasets are lacking, which merits further investigation.

## Conclusions

Five radiomics features and six clinical risk factors were identified to be associated with postsurgical recurrence of PTC. The combined model may have potential for better predicting PTC recurrence than radiomics and clinical models alone. Further testing with larger cohort may help reach statistical significance.

### Electronic supplementary material

Below is the link to the electronic supplementary material.

Supplementary Material 1

Supplementary Material 2

## Data Availability

The datasets used and analysed during the current study are available from the corresponding author on reasonable request.

## Abbreviations

AUC: Area under the receiver operating characteristic curve
CT: Computed tomography
DCA: Decision curve analysis
DSC: Dice similarity coefficient
ETE: Extrathyroidal extension
GLCM: Grey-Level Co-occurrence Matrix
KNN: K-nearest neighbor
LASSO: Least absolute shrinkage and selection operator
LN: Lymph nodes
LR: Logistic regression
NN: Neural network
PTC: Papillary thyroid carcinoma
ROC: Receiver operating characteristic
SVM: Support vector machine
