# Supplementary material for: Analysis of preoperative computed tomography radiomics and clinical factors for predicting postsurgical recurrence of papillary thyroid carcinoma
Source: Cancer Imaging. 2023 Dec 14;23:118. doi: 10.1186/s40644-023-00629-9 (PMC10722708; doi:10.1186/s40644-023-00629-9)
Supplement: Supplementary file 1 — Supplementary Material 1 [file 40644_2023_629_MOESM1_ESM.docx]

Additional file 1. The imaging and post-processing protocols of three different multi-slice spiral CT scanners.

| Parameter | GE Discovery CT750 HD | GE Optima CT660 | GE Lightspeed VCT |
| --- | --- | --- | --- |
| Number of channels | 64 | 64 | 64 |
| Section collimation | 64 × 0.625 mm | 64 × 0.625 mm | 64 × 0.625 mm |
| Thickness (mm) | 5 | 5 | 5 |
| Interval (mm) | 5 | 5 | 5 |
| Helical pitch | 0.984 | 0.984 | 0.984 |
| Gantry rotation time (s) | 0.5 | 0.8 | 0.5 |
| Tube voltage (kVp) | 120 | 120 | 120 |
