# Supplementary material for: Analysis of preoperative computed tomography radiomics and clinical factors for predicting postsurgical recurrence of papillary thyroid carcinoma
Source: Cancer Imaging. 2023 Dec 14;23:118. doi: 10.1186/s40644-023-00629-9 (PMC10722708; doi:10.1186/s40644-023-00629-9)
Supplement: Supplementary file 2 — Supplementary Material 2 [file 40644_2023_629_MOESM2_ESM.docx]

**A total of 1218 radiomics features initially extracted are listed as follows:**

1. **Shape-based features**

original-shape-elongation

original-shape-flatness

original-shape-least axis length

original-shape-major axis length

original-shape-maximum 2D diameter column

original-shape-maximum 2D diameter row

original-shape-maximum 2D diameter slice

original-shape-maximum 3D diameter

original-shape-mesh volume

original-shape-minor axis length

original-shape-sphericity

original-shape-surface area

original-shape-surface volume ratio

original-shape-voxel volume

1. **First-order features**

original-firstorder-10 percentile

original-firstorder-90 percentile

original-firstorder-energy

original-firstorder-entropy

original-firstorder-interquartile range

original-firstorder-kurtosis

original-firstorder-maximum

original-firstorder-mean

original-firstorder-mean absolute deviation

original-firstorder-median

original-firstorder-minimum

original-firstorder-range

original-firstorder-robust mean absolute deviation

original-firstorder-root mean squared

original-firstorder-skewness

original-firstorder-total energy

original-firstorder-uniformity

original-firstorder-variance

log-sigma-1-0-mm-3D-firstorder-10 percentile

log-sigma-1-0-mm-3D-firstorder-90 percentile

log-sigma-1-0-mm-3D-firstorder-energy

log-sigma-1-0-mm-3D-firstorder-entropy

log-sigma-1-0-mm-3D-firstorder-interquartile range

log-sigma-1-0-mm-3D-firstorder-kurtosis

log-sigma-1-0-mm-3D-firstorder-maximum

log-sigma-1-0-mm-3D-firstorder-mean

log-sigma-1-0-mm-3D-firstorder-mean absolute deviation

log-sigma-1-0-mm-3D-firstorder-median

log-sigma-1-0-mm-3D-firstorder-minimum

log-sigma-1-0-mm-3D-firstorder-range

log-sigma-1-0-mm-3D-firstorder-robust mean absolute deviation

log-sigma-1-0-mm-3D-firstorder-root mean squared

log-sigma-1-0-mm-3D-firstorder-skewness

log-sigma-1-0-mm-3D-firstorder-total energy

log-sigma-1-0-mm-3D-firstorder-uniformity

log-sigma-1-0-mm-3D-firstorder-variance

log-sigma-2-0-mm-3D-firstorder-10 percentile

log-sigma-2-0-mm-3D-firstorder-90 percentile

log-sigma-2-0-mm-3D-firstorder-energy

log-sigma-2-0-mm-3D-firstorder-entropy

log-sigma-2-0-mm-3D-firstorder-interquartile range

log-sigma-2-0-mm-3D-firstorder-kurtosis

log-sigma-2-0-mm-3D-firstorder-maximum

log-sigma-2-0-mm-3D-firstorder-mean

log-sigma-2-0-mm-3D-firstorder-mean absolute deviation

log-sigma-2-0-mm-3D-firstorder-median

log-sigma-2-0-mm-3D-firstorder-minimum

log-sigma-2-0-mm-3D-firstorder-range

log-sigma-2-0-mm-3D-firstorder-robust mean absolute deviation

log-sigma-2-0-mm-3D-firstorder-root mean squared

log-sigma-2-0-mm-3D-firstorder-skewness

log-sigma-2-0-mm-3D-firstorder-total energy

log-sigma-2-0-mm-3D-firstorder-uniformity

log-sigma-2-0-mm-3D-firstorder-variance

log-sigma-3-0-mm-3D-firstorder-10 percentile

log-sigma-3-0-mm-3D-firstorder-90 percentile

log-sigma-3-0-mm-3D-firstorder-energy

log-sigma-3-0-mm-3D-firstorder-entropy

log-sigma-3-0-mm-3D-firstorder-interquartile range

log-sigma-3-0-mm-3D-firstorder-kurtosis

log-sigma-3-0-mm-3D-firstorder-maximum

log-sigma-3-0-mm-3D-firstorder-mean

log-sigma-3-0-mm-3D-firstorder-mean absolute deviation

log-sigma-3-0-mm-3D-firstorder-median

log-sigma-3-0-mm-3D-firstorder-minimum

log-sigma-3-0-mm-3D-firstorder-range

log-sigma-3-0-mm-3D-firstorder-robust mean absolute deviation

log-sigma-3-0-mm-3D-firstorder-root mean squared

log-sigma-3-0-mm-3D-firstorder-skewness

log-sigma-3-0-mm-3D-firstorder-total energy

log-sigma-3-0-mm-3D-firstorder-uniformity

log-sigma-3-0-mm-3D-firstorder-variance

log-sigma-4-0-mm-3D-firstorder-10 percentile

log-sigma-4-0-mm-3D-firstorder-90 percentile

log-sigma-4-0-mm-3D-firstorder-energy

log-sigma-4-0-mm-3D-firstorder-entropy

log-sigma-4-0-mm-3D-firstorder-interquartile range

log-sigma-4-0-mm-3D-firstorder-kurtosis

log-sigma-4-0-mm-3D-firstorder-maximum

log-sigma-4-0-mm-3D-firstorder-mean

log-sigma-4-0-mm-3D-firstorder-mean absolute deviation

log-sigma-4-0-mm-3D-firstorder-median

log-sigma-4-0-mm-3D-firstorder-minimum

log-sigma-4-0-mm-3D-firstorder-range

log-sigma-4-0-mm-3D-firstorder-robust mean absolute deviation

log-sigma-4-0-mm-3D-firstorder-root mean squared

log-sigma-4-0-mm-3D-firstorder-skewness

log-sigma-4-0-mm-3D-firstorder-total energy

log-sigma-4-0-mm-3D-firstorder-uniformity

log-sigma-4-0-mm-3D-firstorder-variance

log-sigma-5-0-mm-3D-firstorder-10 percentile

log-sigma-5-0-mm-3D-firstorder-90 percentile

log-sigma-5-0-mm-3D-firstorder-energy

log-sigma-5-0-mm-3D-firstorder-entropy

log-sigma-5-0-mm-3D-firstorder-interquartile range

log-sigma-5-0-mm-3D-firstorder-kurtosis

log-sigma-5-0-mm-3D-firstorder-maximum

log-sigma-5-0-mm-3D-firstorder-mean

log-sigma-5-0-mm-3D-firstorder-mean absolute deviation

log-sigma-5-0-mm-3D-firstorder-median

log-sigma-5-0-mm-3D-firstorder-minimum

log-sigma-5-0-mm-3D-firstorder-range

log-sigma-5-0-mm-3D-firstorder-robust mean absolute deviation

log-sigma-5-0-mm-3D-firstorder-root mean squared

log-sigma-5-0-mm-3D-firstorder-skewness

log-sigma-5-0-mm-3D-firstorder-total energy

log-sigma-5-0-mm-3D-firstorder-uniformity

log-sigma-5-0-mm-3D-firstorder-variance

wavelet-HHL-firstorder-10 percentile

wavelet-HHL-firstorder-90 percentile

wavelet-HHL-firstorder-energy

wavelet-HHL-firstorder-entropy

wavelet-HHL-firstorder-interquartile range

wavelet-HHL-firstorder-kurtosis

wavelet-HHL-firstorder-maximum

wavelet-HHL-firstorder-mean

wavelet-HHL-firstorder-mean absolute deviation

wavelet-HHL-firstorder-median

wavelet-HHL-firstorder-minimum

wavelet-HHL-firstorder-range

wavelet-HHL-firstorder-robust mean absolute deviation

wavelet-HHL-firstorder-root mean squared

wavelet-HHL-firstorder-skewness

wavelet-HHL-firstorder-total energy

wavelet-HHL-firstorder-uniformity

wavelet-HHL-firstorder-variance

wavelet-HHH-firstorder-10 percentile

wavelet-HHH-firstorder-90 percentile

wavelet-HHH-firstorder-energy

wavelet-HHH-firstorder-entropy

wavelet-HHH-firstorder-interquartile range

wavelet-HHH-firstorder-kurtosis

wavelet-HHH-firstorder-maximum

wavelet-HHH-firstorder-mean

wavelet-HHH-firstorder-mean absolute deviation

wavelet-HHH-firstorder-median

wavelet-HHH-firstorder-minimum

wavelet-HHH-firstorder-range

wavelet-HHH-firstorder-robust mean absolute deviation

wavelet-HHH-firstorder-root mean squared

wavelet-HHH-firstorder-skewness

wavelet-HHH-firstorder-total energy

wavelet-HHH-firstorder-uniformity

wavelet-HHH-firstorder-variance

wavelet-HLH-firstorder-10 percentile

wavelet-HLH-firstorder-90 percentile

wavelet-HLH-firstorder-energy

wavelet-HLH-firstorder-entropy

wavelet-HLH-firstorder-interquartile range

wavelet-HLH-firstorder-kurtosis

wavelet-HLH-firstorder-maximum

wavelet-HLH-firstorder-mean

wavelet-HLH-firstorder-mean absolute deviation

wavelet-HLH-firstorder-median

wavelet-HLH-firstorder-minimum

wavelet-HLH-firstorder-range

wavelet-HLH-firstorder-robust mean absolute deviation

wavelet-HLH-firstorder-root mean squared

wavelet-HLH-firstorder-skewness

wavelet-HLH-firstorder-total energy

wavelet-HLH-firstorder-uniformity

wavelet-HLH-firstorder-variance

wavelet-HLL-firstorder-10 percentile

wavelet-HLL-firstorder-90 percentile

wavelet-HLL-firstorder-energy

wavelet-HLL-firstorder-entropy

wavelet-HLL-firstorder-interquartile range

wavelet-HLL-firstorder-kurtosis

wavelet-HLL-firstorder-maximum

wavelet-HLL-firstorder-mean

wavelet-HLL-firstorder-mean absolute deviation

wavelet-HLL-firstorder-median

wavelet-HLL-firstorder-minimum

wavelet-HLL-firstorder-range

wavelet-HLL-firstorder-robust mean absolute deviation

wavelet-HLL-firstorder-root mean squared

wavelet-HLL-firstorder-skewness

wavelet-HLL-firstorder-total energy

wavelet-HLL-firstorder-uniformity

wavelet-HLL-firstorder-variance

wavelet-LHH-firstorder-10 percentile

wavelet-LHH-firstorder-90 percentile

wavelet-LHH-firstorder-energy

wavelet-LHH-firstorder-entropy

wavelet-LHH-firstorder-interquartile range

wavelet-LHH-firstorder-kurtosis

wavelet-LHH-firstorder-maximum

wavelet-LHH-firstorder-mean

wavelet-LHH-firstorder-mean absolute deviation

wavelet-LHH-firstorder-median

wavelet-LHH-firstorder-minimum

wavelet-LHH-firstorder-range

wavelet-LHH-firstorder-robust mean absolute deviation

wavelet-LHH-firstorder-root mean squared

wavelet-LHH-firstorder-skewness

wavelet-LHH-firstorder-total energy

wavelet-LHH-firstorder-uniformity

wavelet-LHH-firstorder-variance

wavelet-LHL-firstorder-10 percentile

wavelet-LHL-firstorder-90 percentile

wavelet-LHL-firstorder-energy

wavelet-LHL-firstorder-entropy

wavelet-LHL-firstorder-interquartile range

wavelet-LHL-firstorder-kurtosis

wavelet-LHL-firstorder-maximum

wavelet-LHL-firstorder-mean

wavelet-LHL-firstorder-mean absolute deviation

wavelet-LHL-firstorder-median

wavelet-LHL-firstorder-minimum

wavelet-LHL-firstorder-range

wavelet-LHL-firstorder-robust mean absolute deviation

wavelet-LHL-firstorder-root mean squared

wavelet-LHL-firstorder-skewness

wavelet-LHL-firstorder-total energy

wavelet-LHL-firstorder-uniformity

wavelet-LHL-firstorder-variance

wavelet-LLH-firstorder-10 percentile

wavelet-LLH-firstorder-90 percentile

wavelet-LLH-firstorder-energy

wavelet-LLH-firstorder-entropy

wavelet-LLH-firstorder-interquartile range

wavelet-LLH-firstorder-kurtosis

wavelet-LLH-firstorder-maximum

wavelet-LLH-firstorder-mean

wavelet-LLH-firstorder-mean absolute deviation

wavelet-LLH-firstorder-median

wavelet-LLH-firstorder-minimum

wavelet-LLH-firstorder-range

wavelet-LLH-firstorder-robust mean absolute deviation

wavelet-LLH-firstorder-root mean squared

wavelet-LLH-firstorder-skewness

wavelet-LLH-firstorder-total energy

wavelet-LLH-firstorder-uniformity

wavelet-LLH-firstorder-variance

wavelet-LLL-firstorder-10 percentile

wavelet-LLL-firstorder-90 percentile

wavelet-LLL-firstorder-energy

wavelet-LLL-firstorder-entropy

wavelet-LLL-firstorder-interquartile range

wavelet-LLL-firstorder-kurtosis

wavelet-LLL-firstorder-maximum

wavelet-LLL-firstorder-mean

wavelet-LLL-firstorder-mean absolute deviation

wavelet-LLL-firstorder-median

wavelet-LLL-firstorder-minimum

wavelet-LLL-firstorder-range

wavelet-LLL-firstorder-robust mean absolute deviation

wavelet-LLL-firstorder-root mean squared

wavelet-LLL-firstorder-skewness

wavelet-LLL-firstorder-total energy

wavelet-LLL-firstorder-uniformity

wavelet-LLL-firstorder-variance

1. **Texture features**

original-GLCM-autocorrelation

original-GLCM-cluster prominence

original-GLCM-cluster shade

original-GLCM-cluster tendency

original-GLCM-contrast

original-GLCM-correlation

original-GLCM-difference average

original-GLCM-difference entropy

original-GLCM-difference variance

original-GLCM-inverse difference

original-GLCM-inverse difference moment

original-GLCM-inverse difference moment normalized

original-GLCM-inverse difference normalized

original-GLCM-informational measure of correlation 1

original-GLCM-informational measure of correlation 2

original-GLCM-inverse variance

original-GLCM-joint average

original-GLCM-joint energy

original-GLCM-joint entropy

original-GLCM-maximum probability

original-GLCM-sum entropy

original-GLCM-sum squares

log-sigma-1-0-mm-3D-GLCM-autocorrelation

log-sigma-1-0-mm-3D-GLCM-cluster prominence

log-sigma-1-0-mm-3D-GLCM-cluster shade

log-sigma-1-0-mm-3D-GLCM-cluster tendency

log-sigma-1-0-mm-3D-GLCM-contrast

log-sigma-1-0-mm-3D-GLCM-correlation

log-sigma-1-0-mm-3D-GLCM-difference average

log-sigma-1-0-mm-3D-GLCM-difference entropy

log-sigma-1-0-mm-3D-GLCM-difference variance

log-sigma-1-0-mm-3D-GLCM-inverse difference

log-sigma-1-0-mm-3D-GLCM-inverse difference moment

log-sigma-1-0-mm-3D-GLCM-inverse difference moment normalized

log-sigma-1-0-mm-3D-GLCM-inverse difference normalized

log-sigma-1-0-mm-3D-GLCM-informational measure of correlation 1

log-sigma-1-0-mm-3D-GLCM-informational measure of correlation 2

log-sigma-1-0-mm-3D-GLCM-inverse variance

log-sigma-1-0-mm-3D-GLCM-joint average

log-sigma-1-0-mm-3D-GLCM-joint energy

log-sigma-1-0-mm-3D-GLCM-joint entropy

log-sigma-1-0-mm-3D-GLCM-maximum probability

log-sigma-1-0-mm-3D-GLCM-sum entropy

log-sigma-1-0-mm-3D-GLCM-sum squares

log-sigma-2-0-mm-3D-GLCM-autocorrelation

log-sigma-2-0-mm-3D-GLCM-cluster prominence

log-sigma-2-0-mm-3D-GLCM-cluster shade

log-sigma-2-0-mm-3D-GLCM-cluster tendency

log-sigma-2-0-mm-3D-GLCM-contrast

log-sigma-2-0-mm-3D-GLCM-correlation

log-sigma-2-0-mm-3D-GLCM-difference average

log-sigma-2-0-mm-3D-GLCM-difference entropy

log-sigma-2-0-mm-3D-GLCM-difference variance

log-sigma-2-0-mm-3D-GLCM-inverse difference

log-sigma-2-0-mm-3D-GLCM-inverse difference moment

log-sigma-2-0-mm-3D-GLCM-inverse difference moment normalized

log-sigma-2-0-mm-3D-GLCM-inverse difference normalized

log-sigma-2-0-mm-3D-GLCM-informational measure of correlation 1

log-sigma-2-0-mm-3D-GLCM-informational measure of correlation 2

log-sigma-2-0-mm-3D-GLCM-inverse variance

log-sigma-2-0-mm-3D-GLCM-joint average

log-sigma-2-0-mm-3D-GLCM-joint energy

log-sigma-2-0-mm-3D-GLCM-joint entropy

log-sigma-2-0-mm-3D-GLCM-maximum probability

log-sigma-2-0-mm-3D-GLCM-sum entropy

log-sigma-2-0-mm-3D-GLCM-sum squares

log-sigma-3-0-mm-3D-GLCM-autocorrelation

log-sigma-3-0-mm-3D-GLCM-cluster prominence

log-sigma-3-0-mm-3D-GLCM-cluster shade

log-sigma-3-0-mm-3D-GLCM-cluster tendency

log-sigma-3-0-mm-3D-GLCM-contrast

log-sigma-3-0-mm-3D-GLCM-correlation

log-sigma-3-0-mm-3D-GLCM-difference average

log-sigma-3-0-mm-3D-GLCM-difference entropy

log-sigma-3-0-mm-3D-GLCM-difference variance

log-sigma-3-0-mm-3D-GLCM-inverse difference

log-sigma-3-0-mm-3D-GLCM-inverse difference moment

log-sigma-3-0-mm-3D-GLCM-inverse difference moment normalized

log-sigma-3-0-mm-3D-GLCM-inverse difference normalized

log-sigma-3-0-mm-3D-GLCM-informational measure of correlation 1

log-sigma-3-0-mm-3D-GLCM-informational measure of correlation 2

log-sigma-3-0-mm-3D-GLCM-inverse variance

log-sigma-3-0-mm-3D-GLCM-joint average

log-sigma-3-0-mm-3D-GLCM-joint energy

log-sigma-3-0-mm-3D-GLCM-joint entropy

log-sigma-3-0-mm-3D-GLCM-maximum probability

log-sigma-3-0-mm-3D-GLCM-sum entropy

log-sigma-3-0-mm-3D-GLCM-sum squares

log-sigma-4-0-mm-3D-GLCM-autocorrelation

log-sigma-4-0-mm-3D-GLCM-cluster prominence

log-sigma-4-0-mm-3D-GLCM-cluster shade

log-sigma-4-0-mm-3D-GLCM-cluster tendency

log-sigma-4-0-mm-3D-GLCM-contrast

log-sigma-4-0-mm-3D-GLCM-correlation

log-sigma-4-0-mm-3D-GLCM-difference average

log-sigma-4-0-mm-3D-GLCM-difference entropy

log-sigma-4-0-mm-3D-GLCM-difference variance

log-sigma-4-0-mm-3D-GLCM-inverse difference

log-sigma-4-0-mm-3D-GLCM-inverse difference moment

log-sigma-4-0-mm-3D-GLCM-inverse difference moment normalized

log-sigma-4-0-mm-3D-GLCM-inverse difference normalized

log-sigma-4-0-mm-3D-GLCM-informational measure of correlation 1

log-sigma-4-0-mm-3D-GLCM-informational measure of correlation 2

log-sigma-4-0-mm-3D-GLCM-inverse variance

log-sigma-4-0-mm-3D-GLCM-joint average

log-sigma-4-0-mm-3D-GLCM-joint energy

log-sigma-4-0-mm-3D-GLCM-joint entropy

log-sigma-4-0-mm-3D-GLCM-maximum probability

log-sigma-4-0-mm-3D-GLCM-sum entropy

log-sigma-4-0-mm-3D-GLCM-sum squares

log-sigma-5-0-mm-3D-GLCM-autocorrelation

log-sigma-5-0-mm-3D-GLCM-cluster prominence

log-sigma-5-0-mm-3D-GLCM-cluster shade

log-sigma-5-0-mm-3D-GLCM-cluster tendency

log-sigma-5-0-mm-3D-GLCM-contrast

log-sigma-5-0-mm-3D-GLCM-correlation

log-sigma-5-0-mm-3D-GLCM-difference average

log-sigma-5-0-mm-3D-GLCM-difference entropy

log-sigma-5-0-mm-3D-GLCM-difference variance

log-sigma-5-0-mm-3D-GLCM-inverse difference

log-sigma-5-0-mm-3D-GLCM-inverse difference moment

log-sigma-5-0-mm-3D-GLCM-inverse difference moment normalized

log-sigma-5-0-mm-3D-GLCM-inverse difference normalized

log-sigma-5-0-mm-3D-GLCM-informational measure of correlation 1

log-sigma-5-0-mm-3D-GLCM-informational measure of correlation 2

log-sigma-5-0-mm-3D-GLCM-inverse variance

log-sigma-5-0-mm-3D-GLCM-joint average

log-sigma-5-0-mm-3D-GLCM-joint energy

log-sigma-5-0-mm-3D-GLCM-joint entropy

log-sigma-5-0-mm-3D-GLCM-maximum probability

log-sigma-5-0-mm-3D-GLCM-sum entropy

log-sigma-5-0-mm-3D-GLCM-sum squares

wavelet-HHH-GLCM-autocorrelation

wavelet-HHH-GLCM-cluster prominence

wavelet-HHH-GLCM-cluster shade

wavelet-HHH-GLCM-cluster tendency

wavelet-HHH-GLCM-contrast

wavelet-HHH-GLCM-correlation

wavelet-HHH-GLCM-difference average

wavelet-HHH-GLCM-difference entropy

wavelet-HHH-GLCM-difference variance

wavelet-HHH-GLCM-inverse difference

wavelet-HHH-GLCM-inverse difference moment

wavelet-HHH-GLCM-inverse difference moment normalized

wavelet-HHH-GLCM-inverse difference normalized

wavelet-HHH-GLCM-informational measure of correlation 1

wavelet-HHH-GLCM-informational measure of correlation 2

wavelet-HHH-GLCM-inverse variance

wavelet-HHH-GLCM-joint average

wavelet-HHH-GLCM-joint energy

wavelet-HHH-GLCM-joint entropy

wavelet-HHH-GLCM-maximum probability

wavelet-HHH-GLCM-sum entropy

wavelet-HHH-GLCM-sum squares

wavelet-HHL-GLCM-autocorrelation

wavelet-HHL-GLCM-cluster prominence

wavelet-HHL-GLCM-cluster shade

wavelet-HHL-GLCM-cluster tendency

wavelet-HHL-GLCM-contrast

wavelet-HHL-GLCM-correlation

wavelet-HHL-GLCM-difference average

wavelet-HHL-GLCM-difference entropy

wavelet-HHL-GLCM-difference variance

wavelet-HHL-GLCM-inverse difference

wavelet-HHL-GLCM-inverse difference moment

wavelet-HHL-GLCM-inverse difference moment normalized

wavelet-HHL-GLCM-inverse difference normalized

wavelet-HHL-GLCM-informational measure of correlation 1

wavelet-HHL-GLCM-informational measure of correlation 2

wavelet-HHL-GLCM-inverse variance

wavelet-HHL-GLCM-joint average

wavelet-HHL-GLCM-joint energy

wavelet-HHL-GLCM-joint entropy

wavelet-HHL-GLCM-maximum probability

wavelet-HHL-GLCM-sum entropy

wavelet-HHL-GLCM-sum squares

wavelet-HLH-GLCM-autocorrelation

wavelet-HLH-GLCM-cluster prominence

wavelet-HLH-GLCM-cluster shade

wavelet-HLH-GLCM-cluster tendency

wavelet-HLH-GLCM-contrast

wavelet-HLH-GLCM-correlation

wavelet-HLH-GLCM-difference average

wavelet-HLH-GLCM-difference entropy

wavelet-HLH-GLCM-difference variance

wavelet-HLH-GLCM-inverse difference

wavelet-HLH-GLCM-inverse difference moment

wavelet-HLH-GLCM-inverse difference moment normalized

wavelet-HLH-GLCM-inverse difference normalized

wavelet-HLH-GLCM-informational measure of correlation 1

wavelet-HLH-GLCM-informational measure of correlation 2

wavelet-HLH-GLCM-inverse variance

wavelet-HLH-GLCM-joint average

wavelet-HLH-GLCM-joint energy

wavelet-HLH-GLCM-joint entropy

wavelet-HLH-GLCM-maximum probability

wavelet-HLH-GLCM-sum entropy

wavelet-HLH-GLCM-sum squares

wavelet-HLL-GLCM-autocorrelation

wavelet-HLL-GLCM-cluster prominence

wavelet-HLL-GLCM-cluster shade

wavelet-HLL-GLCM-cluster tendency

wavelet-HLL-GLCM-contrast

wavelet-HLL-GLCM-correlation

wavelet-HLL-GLCM-difference average

wavelet-HLL-GLCM-difference entropy

wavelet-HLL-GLCM-difference variance

wavelet-HLL-GLCM-inverse difference

wavelet-HLL-GLCM-inverse difference moment

wavelet-HLL-GLCM-inverse difference moment normalized

wavelet-HLL-GLCM-inverse difference normalized

wavelet-HLL-GLCM-informational measure of correlation 1

wavelet-HLL-GLCM-informational measure of correlation 2

wavelet-HLL-GLCM-inverse variance

wavelet-HLL-GLCM-joint average

wavelet-HLL-GLCM-joint energy

wavelet-HLL-GLCM-joint entropy

wavelet-HLL-GLCM-maximum probability

wavelet-HLL-GLCM-sum entropy

wavelet-HLL-GLCM-sum squares

wavelet-LHH-GLCM-autocorrelation

wavelet-LHH-GLCM-cluster prominence

wavelet-LHH-GLCM-cluster shade

wavelet-LHH-GLCM-cluster tendency

wavelet-LHH-GLCM-contrast

wavelet-LHH-GLCM-correlation

wavelet-LHH-GLCM-difference average

wavelet-LHH-GLCM-difference entropy

wavelet-LHH-GLCM-difference variance

wavelet-LHH-GLCM-inverse difference

wavelet-LHH-GLCM-inverse difference moment

wavelet-LHH-GLCM-inverse difference moment normalized

wavelet-LHH-GLCM-inverse difference normalized

wavelet-LHH-GLCM-informational measure of correlation 1

wavelet-LHH-GLCM-informational measure of correlation 2

wavelet-LHH-GLCM-inverse variance

wavelet-LHH-GLCM-joint average

wavelet-LHH-GLCM-joint energy

wavelet-LHH-GLCM-joint entropy

wavelet-LHH-GLCM-maximum probability

wavelet-LHH-GLCM-sum entropy

wavelet-LHH-GLCM-sum squares

wavelet-LHL-GLCM-autocorrelation

wavelet-LHL-GLCM-cluster prominence

wavelet-LHL-GLCM-cluster shade

wavelet-LHL-GLCM-cluster tendency

wavelet-LHL-GLCM-contrast

wavelet-LHL-GLCM-correlation

wavelet-LHL-GLCM-difference average

wavelet-LHL-GLCM-difference entropy

wavelet-LHL-GLCM-difference variance

wavelet-LHL-GLCM-inverse difference

wavelet-LHL-GLCM-inverse difference moment

wavelet-LHL-GLCM-inverse difference moment normalized

wavelet-LHL-GLCM-inverse difference normalized

wavelet-LHL-GLCM-informational measure of correlation 1

wavelet-LHL-GLCM-informational measure of correlation 2

wavelet-LHL-GLCM-inverse variance

wavelet-LHL-GLCM-joint average

wavelet-LHL-GLCM-joint energy

wavelet-LHL-GLCM-joint entropy

wavelet-LHL-GLCM-maximum probability

wavelet-LHL-GLCM-sum entropy

wavelet-LHL-GLCM-sum squares

wavelet-LLH-GLCM-autocorrelation

wavelet-LLH-GLCM-cluster prominence

wavelet-LLH-GLCM-cluster shade

wavelet-LLH-GLCM-cluster tendency

wavelet-LLH-GLCM-contrast

wavelet-LLH-GLCM-correlation

wavelet-LLH-GLCM-difference average

wavelet-LLH-GLCM-difference entropy

wavelet-LLH-GLCM-difference variance

wavelet-LLH-GLCM-inverse difference

wavelet-LLH-GLCM-inverse difference moment

wavelet-LLH-GLCM-inverse difference moment normalized

wavelet-LLH-GLCM-inverse difference normalized

wavelet-LLH-GLCM-informational measure of correlation 1

wavelet-LLH-GLCM-informational measure of correlation 2

wavelet-LLH-GLCM-inverse variance

wavelet-LLH-GLCM-joint average

wavelet-LLH-GLCM-joint energy

wavelet-LLH-GLCM-joint entropy

wavelet-LLH-GLCM-maximum probability

wavelet-LLH-GLCM-sum entropy

wavelet-LLH-GLCM-sum squares

wavelet-LLL-GLCM-autocorrelation

wavelet-LLL-GLCM-cluster prominence

wavelet-LLL-GLCM-cluster shade

wavelet-LLL-GLCM-cluster tendency

wavelet-LLL-GLCM-contrast

wavelet-LLL-GLCM-correlation

wavelet-LLL-GLCM-difference average

wavelet-LLL-GLCM-difference entropy

wavelet-LLL-GLCM-difference variance

wavelet-LLL-GLCM-inverse difference

wavelet-LLL-GLCM-inverse difference moment

wavelet-LLL-GLCM-inverse difference moment normalized

wavelet-LLL-GLCM-inverse difference normalized

wavelet-LLL-GLCM-informational measure of correlation 1

wavelet-LLL-GLCM-informational measure of correlation 2

wavelet-LLL-GLCM-inverse variance

wavelet-LLL-GLCM-joint average

wavelet-LLL-GLCM-joint energy

wavelet-LLL-GLCM-joint entropy

wavelet-LLL-GLCM-maximum probability

wavelet-LLL-GLCM-sum entropy

wavelet-LLL-GLCM-sum squares

original-GLDM-dependence entropy

original-GLDM-dependence nonuniformity

original-GLDM-dependence nonuniformity normalized

original-GLDM-dependence variance

original-GLDM-gray level nonuniformity

original-GLDM-gray level variance

original-GLDM-high gray level emphasis

original-GLDM-large dependence emphasis

original-GLDM-large dependence high gray level emphasis

original-GLDM-large dependence low gray level emphasis

original-GLDM-low gray level emphasis

original-GLDM-small dependence emphasis

original-GLDM-small dependence high gray level emphasis

original-GLDM-small dependence low gray level emphasis

log-sigma-1-0-mm-3D-GLDM-dependence entropy

log-sigma-1-0-mm-3D-GLDM-dependence nonuniformity

log-sigma-1-0-mm-3D-GLDM-dependence nonuniformity normalized

log-sigma-1-0-mm-3D-GLDM-dependence variance

log-sigma-1-0-mm-3D-GLDM-gray level nonuniformity

log-sigma-1-0-mm-3D-GLDM-gray level variance

log-sigma-1-0-mm-3D-GLDM-high gray level emphasis

log-sigma-1-0-mm-3D-GLDM-large dependence emphasis

log-sigma-1-0-mm-3D-GLDM-large dependence high gray level emphasis

log-sigma-1-0-mm-3D-GLDM-large dependence low gray level emphasis

log-sigma-1-0-mm-3D-GLDM-low gray level emphasis

log-sigma-1-0-mm-3D-GLDM-small dependence emphasis

log-sigma-1-0-mm-3D-GLDM-small dependence high gray level emphasis

log-sigma-1-0-mm-3D-GLDM-small dependence low gray level emphasis

log-sigma-2-0-mm-3D-GLDM-dependence entropy

log-sigma-2-0-mm-3D-GLDM-dependence nonuniformity

log-sigma-2-0-mm-3D-GLDM-dependence nonuniformity normalized

log-sigma-2-0-mm-3D-GLDM-dependence variance

log-sigma-2-0-mm-3D-GLDM-gray level nonuniformity

log-sigma-2-0-mm-3D-GLDM-gray level variance

log-sigma-2-0-mm-3D-GLDM-high gray level emphasis

log-sigma-2-0-mm-3D-GLDM-large dependence emphasis

log-sigma-2-0-mm-3D-GLDM-large dependence high gray level emphasis

log-sigma-2-0-mm-3D-GLDM-large dependence low gray level emphasis

log-sigma-2-0-mm-3D-GLDM-low gray level emphasis

log-sigma-2-0-mm-3D-GLDM-small dependence emphasis

log-sigma-2-0-mm-3D-GLDM-small dependence high gray level emphasis

log-sigma-2-0-mm-3D-GLDM-small dependence low gray level emphasis

log-sigma-3-0-mm-3D-GLDM-dependence entropy

log-sigma-3-0-mm-3D-GLDM-dependence nonuniformity

log-sigma-3-0-mm-3D-GLDM-dependence nonuniformity normalized

log-sigma-3-0-mm-3D-GLDM-dependence variance

log-sigma-3-0-mm-3D-GLDM-gray level nonuniformity

log-sigma-3-0-mm-3D-GLDM-gray level variance

log-sigma-3-0-mm-3D-GLDM-high gray level emphasis

log-sigma-3-0-mm-3D-GLDM-large dependence emphasis

log-sigma-3-0-mm-3D-GLDM-large dependence high gray level emphasis

log-sigma-3-0-mm-3D-GLDM-large dependence low gray level emphasis

log-sigma-3-0-mm-3D-GLDM-low gray level emphasis

log-sigma-3-0-mm-3D-GLDM-small dependence emphasis

log-sigma-3-0-mm-3D-GLDM-small dependence high gray level emphasis

log-sigma-3-0-mm-3D-GLDM-small dependence low gray level emphasis

log-sigma-4-0-mm-3D-GLDM-dependence entropy

log-sigma-4-0-mm-3D-GLDM-dependence nonuniformity

log-sigma-4-0-mm-3D-GLDM-dependence nonuniformity normalized

log-sigma-4-0-mm-3D-GLDM-dependence variance

log-sigma-4-0-mm-3D-GLDM-gray level nonuniformity

log-sigma-4-0-mm-3D-GLDM-gray level variance

log-sigma-4-0-mm-3D-GLDM-high gray level emphasis

log-sigma-4-0-mm-3D-GLDM-large dependence emphasis

log-sigma-4-0-mm-3D-GLDM-large dependence high gray level emphasis

log-sigma-4-0-mm-3D-GLDM-large dependence low gray level emphasis

log-sigma-4-0-mm-3D-GLDM-low gray level emphasis

log-sigma-4-0-mm-3D-GLDM-small dependence emphasis

log-sigma-4-0-mm-3D-GLDM-small dependence high gray level emphasis

log-sigma-4-0-mm-3D-GLDM-small dependence low gray level emphasis

log-sigma-5-0-mm-3D-GLDM-dependence entropy

log-sigma-5-0-mm-3D-GLDM-dependence nonuniformity

log-sigma-5-0-mm-3D-GLDM-dependence nonuniformity normalized

log-sigma-5-0-mm-3D-GLDM-dependence variance

log-sigma-5-0-mm-3D-GLDM-gray level nonuniformity

log-sigma-5-0-mm-3D-GLDM-gray level variance

log-sigma-5-0-mm-3D-GLDM-high gray level emphasis

log-sigma-5-0-mm-3D-GLDM-large dependence emphasis

log-sigma-5-0-mm-3D-GLDM-large dependence high gray level emphasis

log-sigma-5-0-mm-3D-GLDM-large dependence low gray level emphasis

log-sigma-5-0-mm-3D-GLDM-low gray level emphasis

log-sigma-5-0-mm-3D-GLDM-small dependence emphasis

log-sigma-5-0-mm-3D-GLDM-small dependence high gray level emphasis

log-sigma-5-0-mm-3D-GLDM-small dependence low gray level emphasis

wavelet-HHH-GLDM-dependence entropy

wavelet-HHH-GLDM-dependence nonuniformity

wavelet-HHH-GLDM-dependence nonuniformity normalized

wavelet-HHH-GLDM-dependence variance

wavelet-HHH-GLDM-gray level nonuniformity

wavelet-HHH-GLDM-gray level variance

wavelet-HHH-GLDM-high gray level emphasis

wavelet-HHH-GLDM-large dependence emphasis

wavelet-HHH-GLDM-large dependence high gray level emphasis

wavelet-HHH-GLDM-large dependence low gray level emphasis

wavelet-HHH-GLDM-low gray level emphasis

wavelet-HHH-GLDM-small dependence emphasis

wavelet-HHH-GLDM-small dependence high gray level emphasis

wavelet-HHH-GLDM-small dependence low gray level emphasis

wavelet-HHL-GLDM-dependence entropy

wavelet-HHL-GLDM-dependence nonuniformity

wavelet-HHL-GLDM-dependence nonuniformity normalized

wavelet-HHL-GLDM-dependence variance

wavelet-HHL-GLDM-gray level nonuniformity

wavelet-HHL-GLDM-gray level variance

wavelet-HHL-GLDM-high gray level emphasis

wavelet-HHL-GLDM-large dependence emphasis

wavelet-HHL-GLDM-large dependence high gray level emphasis

wavelet-HHL-GLDM-large dependence low gray level emphasis

wavelet-HHL-GLDM-low gray level emphasis

wavelet-HHL-GLDM-small dependence emphasis

wavelet-HHL-GLDM-small dependence high gray level emphasis

wavelet-HHL-GLDM-small dependence low gray level emphasis

wavelet-HLH-GLDM-dependence entropy

wavelet-HLH-GLDM-dependence nonuniformity

wavelet-HLH-GLDM-dependence nonuniformity normalized

wavelet-HLH-GLDM-dependence variance

wavelet-HLH-GLDM-gray level nonuniformity

wavelet-HLH-GLDM-gray level variance

wavelet-HLH-GLDM-high gray level emphasis

wavelet-HLH-GLDM-large dependence emphasis

wavelet-HLH-GLDM-large dependence high gray level emphasis

wavelet-HLH-GLDM-large dependence low gray level emphasis

wavelet-HLH-GLDM-low gray level emphasis

wavelet-HLH-GLDM-small dependence emphasis

wavelet-HLH-GLDM-small dependence high gray level emphasis

wavelet-HLH-GLDM-small dependence low gray level emphasis

wavelet-HLL-GLDM-dependence entropy

wavelet-HLL-GLDM-dependence nonuniformity

wavelet-HLL-GLDM-dependence nonuniformity normalized

wavelet-HLL-GLDM-dependence variance

wavelet-HLL-GLDM-gray level nonuniformity

wavelet-HLL-GLDM-gray level variance

wavelet-HLL-GLDM-high gray level emphasis

wavelet-HLL-GLDM-large dependence emphasis

wavelet-HLL-GLDM-large dependence high gray level emphasis

wavelet-HLL-GLDM-large dependence low gray level emphasis

wavelet-HLL-GLDM-low gray level emphasis

wavelet-HLL-GLDM-small dependence emphasis

wavelet-HLL-GLDM-small dependence high gray level emphasis

wavelet-HLL-GLDM-small dependence low gray level emphasis

wavelet-LHH-GLDM-dependence entropy

wavelet-LHH-GLDM-dependence nonuniformity

wavelet-LHH-GLDM-dependence nonuniformity normalized

wavelet-LHH-GLDM-dependence variance

wavelet-LHH-GLDM-gray level nonuniformity

wavelet-LHH-GLDM-gray level variance

wavelet-LHH-GLDM-high gray level emphasis

wavelet-LHH-GLDM-large dependence emphasis

wavelet-LHH-GLDM-large dependence high gray level emphasis

wavelet-LHH-GLDM-large dependence low gray level emphasis

wavelet-LHH-GLDM-low gray level emphasis

wavelet-LHH-GLDM-small dependence emphasis

wavelet-LHH-GLDM-small dependence high gray level emphasis

wavelet-LHH-GLDM-small dependence low gray level emphasis

wavelet-LHL-GLDM-dependence entropy

wavelet-LHL-GLDM-dependence nonuniformity

wavelet-LHL-GLDM-dependence nonuniformity normalized

wavelet-LHL-GLDM-dependence variance

wavelet-LHL-GLDM-gray level nonuniformity

wavelet-LHL-GLDM-gray level variance

wavelet-LHL-GLDM-high gray level emphasis

wavelet-LHL-GLDM-large dependence emphasis

wavelet-LHL-GLDM-large dependence high gray level emphasis

wavelet-LHL-GLDM-large dependence low gray level emphasis

wavelet-LHL-GLDM-low gray level emphasis

wavelet-LHL-GLDM-small dependence emphasis

wavelet-LHL-GLDM-small dependence high gray level emphasis

wavelet-LHL-GLDM-small dependence low gray level emphasis

wavelet-LLH-GLDM-dependence entropy

wavelet-LLH-GLDM-dependence nonuniformity

wavelet-LLH-GLDM-dependence nonuniformity normalized

wavelet-LLH-GLDM-dependence variance

wavelet-LLH-GLDM-gray level nonuniformity

wavelet-LLH-GLDM-gray level variance

wavelet-LLH-GLDM-high gray level emphasis

wavelet-LLH-GLDM-large dependence emphasis

wavelet-LLH-GLDM-large dependence high gray level emphasis

wavelet-LLH-GLDM-large dependence low gray level emphasis

wavelet-LLH-GLDM-low gray level emphasis

wavelet-LLH-GLDM-small dependence emphasis

wavelet-LLH-GLDM-small dependence high gray level emphasis

wavelet-LLH-GLDM-small dependence low gray level emphasis

wavelet-LLL-GLDM-dependence entropy

wavelet-LLL-GLDM-dependence nonuniformity

wavelet-LLL-GLDM-dependence nonuniformity normalized

wavelet-LLL-GLDM-dependence variance

wavelet-LLL-GLDM-gray level nonuniformity

wavelet-LLL-GLDM-gray level variance

wavelet-LLL-GLDM-high gray level emphasis

wavelet-LLL-GLDM-large dependence emphasis

wavelet-LLL-GLDM-large dependence high gray level emphasis

wavelet-LLL-GLDM-large dependence low gray level emphasis

wavelet-LLL-GLDM-low gray level emphasis

wavelet-LLL-GLDM-small dependence emphasis

wavelet-LLL-GLDM-small dependence high gray level emphasis

wavelet-LLL-GLDM-small dependence low gray level emphasis

original-GLRLM-gray level nonuniformity

original-GLRLM-gray level nonuniformity normalized

original-GLRLM-gray level variance

original-GLRLM-high gray level run emphasis

original-GLRLM-long run emphasis

original-GLRLM-long run high gray level emphasis

original-GLRLM-long run low gray level emphasis

original-GLRLM-low gray level run emphasis

original-GLRLM-run entropy

original-GLRLM-run length nonuniformity

original-GLRLM-run length nonuniformity normalized

original-GLRLM-run percentage

original-GLRLM-run variance

original-GLRLM-short run emphasis

original-GLRLM-short run high gray level emphasis

original-GLRLM-short run low gray level emphasis

log-sigma-1-0-mm-3D-GLRLM-gray level nonuniformity

log-sigma-1-0-mm-3D-GLRLM-gray level nonuniformity normalized

log-sigma-1-0-mm-3D-GLRLM-gray level variance

log-sigma-1-0-mm-3D-GLRLM-high gray level run emphasis

log-sigma-1-0-mm-3D-GLRLM-long run emphasis

log-sigma-1-0-mm-3D-GLRLM-long run high gray level emphasis

log-sigma-1-0-mm-3D-GLRLM-long run low gray level emphasis

log-sigma-1-0-mm-3D-GLRLM-low gray level run emphasis

log-sigma-1-0-mm-3D-GLRLM-run entropy

log-sigma-1-0-mm-3D-GLRLM-run length nonuniformity

log-sigma-1-0-mm-3D-GLRLM-run length nonuniformity normalized

log-sigma-1-0-mm-3D-GLRLM-run percentage

log-sigma-1-0-mm-3D-GLRLM-run variance

log-sigma-1-0-mm-3D-GLRLM-short run emphasis

log-sigma-1-0-mm-3D-GLRLM-short run high gray level emphasis

log-sigma-1-0-mm-3D-GLRLM-short run low gray level emphasis

log-sigma-2-0-mm-3D-GLRLM-gray level nonuniformity

log-sigma-2-0-mm-3D-GLRLM-gray level nonuniformity normalized

log-sigma-2-0-mm-3D-GLRLM-gray level variance

log-sigma-2-0-mm-3D-GLRLM-high gray level run emphasis

log-sigma-2-0-mm-3D-GLRLM-long run emphasis

log-sigma-2-0-mm-3D-GLRLM-long run high gray level emphasis

log-sigma-2-0-mm-3D-GLRLM-long run low gray level emphasis

log-sigma-2-0-mm-3D-GLRLM-low gray level run emphasis

log-sigma-2-0-mm-3D-GLRLM-run entropy

log-sigma-2-0-mm-3D-GLRLM-run length nonuniformity

log-sigma-2-0-mm-3D-GLRLM-run length nonuniformity normalized

log-sigma-2-0-mm-3D-GLRLM-run percentage

log-sigma-2-0-mm-3D-GLRLM-run variance

log-sigma-2-0-mm-3D-GLRLM-short run emphasis

log-sigma-2-0-mm-3D-GLRLM-short run high gray level emphasis

log-sigma-2-0-mm-3D-GLRLM-short run low gray level emphasis

log-sigma-3-0-mm-3D-GLRLM-gray level nonuniformity

log-sigma-3-0-mm-3D-GLRLM-gray level nonuniformity normalized

log-sigma-3-0-mm-3D-GLRLM-gray level variance

log-sigma-3-0-mm-3D-GLRLM-high gray level run emphasis

log-sigma-3-0-mm-3D-GLRLM-long run emphasis

log-sigma-3-0-mm-3D-GLRLM-long run high gray level emphasis

log-sigma-3-0-mm-3D-GLRLM-long run low gray level emphasis

log-sigma-3-0-mm-3D-GLRLM-low gray level run emphasis

log-sigma-3-0-mm-3D-GLRLM-run entropy

log-sigma-3-0-mm-3D-GLRLM-run length nonuniformity

log-sigma-3-0-mm-3D-GLRLM-run length nonuniformity normalized

log-sigma-3-0-mm-3D-GLRLM-run percentage

log-sigma-3-0-mm-3D-GLRLM-run variance

log-sigma-3-0-mm-3D-GLRLM-short run emphasis

log-sigma-3-0-mm-3D-GLRLM-short run high gray level emphasis

log-sigma-3-0-mm-3D-GLRLM-short run low gray level emphasis

log-sigma-4-0-mm-3D-GLRLM-gray level nonuniformity

log-sigma-4-0-mm-3D-GLRLM-gray level nonuniformity normalized

log-sigma-4-0-mm-3D-GLRLM-gray level variance

log-sigma-4-0-mm-3D-GLRLM-high gray level run emphasis

log-sigma-4-0-mm-3D-GLRLM-long run emphasis

log-sigma-4-0-mm-3D-GLRLM-long run high gray level emphasis

log-sigma-4-0-mm-3D-GLRLM-long run low gray level emphasis

log-sigma-4-0-mm-3D-GLRLM-low gray level run emphasis

log-sigma-4-0-mm-3D-GLRLM-run entropy

log-sigma-4-0-mm-3D-GLRLM-run length nonuniformity

log-sigma-4-0-mm-3D-GLRLM-run length nonuniformity normalized

log-sigma-4-0-mm-3D-GLRLM-run percentage

log-sigma-4-0-mm-3D-GLRLM-run variance

log-sigma-4-0-mm-3D-GLRLM-short run emphasis

log-sigma-4-0-mm-3D-GLRLM-short run high gray level emphasis

log-sigma-4-0-mm-3D-GLRLM-short run low gray level emphasis

log-sigma-5-0-mm-3D-GLRLM-gray level nonuniformity

log-sigma-5-0-mm-3D-GLRLM-gray level nonuniformity normalized

log-sigma-5-0-mm-3D-GLRLM-gray level variance

log-sigma-5-0-mm-3D-GLRLM-high gray level run emphasis

log-sigma-5-0-mm-3D-GLRLM-long run emphasis

log-sigma-5-0-mm-3D-GLRLM-long run high gray level emphasis

log-sigma-5-0-mm-3D-GLRLM-long run low gray level emphasis

log-sigma-5-0-mm-3D-GLRLM-low gray level run emphasis

log-sigma-5-0-mm-3D-GLRLM-run entropy

log-sigma-5-0-mm-3D-GLRLM-run length nonuniformity

log-sigma-5-0-mm-3D-GLRLM-run length nonuniformity normalized

log-sigma-5-0-mm-3D-GLRLM-run percentage

log-sigma-5-0-mm-3D-GLRLM-run variance

log-sigma-5-0-mm-3D-GLRLM-short run emphasis

log-sigma-5-0-mm-3D-GLRLM-short run high gray level emphasis

log-sigma-5-0-mm-3D-GLRLM-short run low gray level emphasis

wavelet-HHH-GLRLM-gray level nonuniformity

wavelet-HHH-GLRLM-gray level nonuniformity normalized

wavelet-HHH-GLRLM-gray level variance

wavelet-HHH-GLRLM-high gray level run emphasis

wavelet-HHH-GLRLM-long run emphasis

wavelet-HHH-GLRLM-long run high gray level emphasis

wavelet-HHH-GLRLM-long run low gray level emphasis

wavelet-HHH-GLRLM-low gray level run emphasis

wavelet-HHH-GLRLM-run entropy

wavelet-HHH-GLRLM-run length nonuniformity

wavelet-HHH-GLRLM-run length nonuniformity normalized

wavelet-HHH-GLRLM-run percentage

wavelet-HHH-GLRLM-run variance

wavelet-HHH-GLRLM-short run emphasis

wavelet-HHH-GLRLM-short run high gray level emphasis

wavelet-HHH-GLRLM-short run low gray level emphasis

wavelet-HHL-GLRLM-gray level nonuniformity

wavelet-HHL-GLRLM-gray level nonuniformity normalized

wavelet-HHL-GLRLM-gray level variance

wavelet-HHL-GLRLM-high gray level run emphasis

wavelet-HHL-GLRLM-long run emphasis

wavelet-HHL-GLRLM-long run high gray level emphasis

wavelet-HHL-GLRLM-long run low gray level emphasis

wavelet-HHL-GLRLM-low gray level run emphasis

wavelet-HHL-GLRLM-run entropy

wavelet-HHL-GLRLM-run length nonuniformity

wavelet-HHL-GLRLM-run length nonuniformity normalized

wavelet-HHL-GLRLM-run percentage

wavelet-HHL-GLRLM-run variance

wavelet-HHL-GLRLM-short run emphasis

wavelet-HHL-GLRLM-short run high gray level emphasis

wavelet-HHL-GLRLM-short run low gray level emphasis

wavelet-HLH-GLRLM-gray level nonuniformity

wavelet-HLH-GLRLM-gray level nonuniformity normalized

wavelet-HLH-GLRLM-gray level variance

wavelet-HLH-GLRLM-high gray level run emphasis

wavelet-HLH-GLRLM-long run emphasis

wavelet-HLH-GLRLM-long run high gray level emphasis

wavelet-HLH-GLRLM-long run low gray level emphasis

wavelet-HLH-GLRLM-low gray level run emphasis

wavelet-HLH-GLRLM-run entropy

wavelet-HLH-GLRLM-run length nonuniformity

wavelet-HLH-GLRLM-run length nonuniformity normalized

wavelet-HLH-GLRLM-run percentage

wavelet-HLH-GLRLM-run variance

wavelet-HLH-GLRLM-short run emphasis

wavelet-HLH-GLRLM-short run high gray level emphasis

wavelet-HLH-GLRLM-short run low gray level emphasis

wavelet-HLL-GLRLM-gray level nonuniformity

wavelet-HLL-GLRLM-gray level nonuniformity normalized

wavelet-HLL-GLRLM-gray level variance

wavelet-HLL-GLRLM-high gray level run emphasis

wavelet-HLL-GLRLM-long run emphasis

wavelet-HLL-GLRLM-long run high gray level emphasis

wavelet-HLL-GLRLM-long run low gray level emphasis

wavelet-HLL-GLRLM-low gray level run emphasis

wavelet-HLL-GLRLM-run entropy

wavelet-HLL-GLRLM-run length nonuniformity

wavelet-HLL-GLRLM-run length nonuniformity normalized

wavelet-HLL-GLRLM-run percentage

wavelet-HLL-GLRLM-run variance

wavelet-HLL-GLRLM-short run emphasis

wavelet-HLL-GLRLM-short run high gray level emphasis

wavelet-HLL-GLRLM-short run low gray level emphasis

wavelet-LHH-GLRLM-gray level nonuniformity

wavelet-LHH-GLRLM-gray level nonuniformity normalized

wavelet-LHH-GLRLM-gray level variance

wavelet-LHH-GLRLM-high gray level run emphasis

wavelet-LHH-GLRLM-long run emphasis

wavelet-LHH-GLRLM-long run high gray level emphasis

wavelet-LHH-GLRLM-long run low gray level emphasis

wavelet-LHH-GLRLM-low gray level run emphasis

wavelet-LHH-GLRLM-run entropy

wavelet-LHH-GLRLM-run length nonuniformity

wavelet-LHH-GLRLM-run length nonuniformity normalized

wavelet-LHH-GLRLM-run percentage

wavelet-LHH-GLRLM-run variance

wavelet-LHH-GLRLM-short run emphasis

wavelet-LHH-GLRLM-short run high gray level emphasis

wavelet-LHH-GLRLM-short run low gray level emphasis

wavelet-LHL-GLRLM-gray level nonuniformity

wavelet-LHL-GLRLM-gray level nonuniformity normalized

wavelet-LHL-GLRLM-gray level variance

wavelet-LHL-GLRLM-high gray level run emphasis

wavelet-LHL-GLRLM-long run emphasis

wavelet-LHL-GLRLM-long run high gray level emphasis

wavelet-LHL-GLRLM-long run low gray level emphasis

wavelet-LHL-GLRLM-low gray level run emphasis

wavelet-LHL-GLRLM-run entropy

wavelet-LHL-GLRLM-run length nonuniformity

wavelet-LHL-GLRLM-run length nonuniformity normalized

wavelet-LHL-GLRLM-run percentage

wavelet-LHL-GLRLM-run variance

wavelet-LHL-GLRLM-short run emphasis

wavelet-LHL-GLRLM-short run high gray level emphasis

wavelet-LHL-GLRLM-short run low gray level emphasis

wavelet-LLH-GLRLM-gray level nonuniformity

wavelet-LLH-GLRLM-gray level nonuniformity normalized

wavelet-LLH-GLRLM-gray level variance

wavelet-LLH-GLRLM-high gray level run emphasis

wavelet-LLH-GLRLM-long run emphasis

wavelet-LLH-GLRLM-long run high gray level emphasis

wavelet-LLH-GLRLM-long run low gray level emphasis

wavelet-LLH-GLRLM-low gray level run emphasis

wavelet-LLH-GLRLM-run entropy

wavelet-LLH-GLRLM-run length nonuniformity

wavelet-LLH-GLRLM-run length nonuniformity normalized

wavelet-LLH-GLRLM-run percentage

wavelet-LLH-GLRLM-run variance

wavelet-LLH-GLRLM-short run emphasis

wavelet-LLH-GLRLM-short run high gray level emphasis

wavelet-LLH-GLRLM-short run low gray level emphasis

wavelet-LLL-GLRLM-gray level nonuniformity

wavelet-LLL-GLRLM-gray level nonuniformity normalized

wavelet-LLL-GLRLM-gray level variance

wavelet-LLL-GLRLM-high gray level run emphasis

wavelet-LLL-GLRLM-long run emphasis

wavelet-LLL-GLRLM-long run high gray level emphasis

wavelet-LLL-GLRLM-long run low gray level emphasis

wavelet-LLL-GLRLM-low gray level run emphasis

wavelet-LLL-GLRLM-run entropy

wavelet-LLL-GLRLM-run length nonuniformity

wavelet-LLL-GLRLM-run length nonuniformity normalized

wavelet-LLL-GLRLM-run percentage

wavelet-LLL-GLRLM-run variance

wavelet-LLL-GLRLM-short run emphasis

wavelet-LLL-GLRLM-short run high gray level emphasis

wavelet-LLL-GLRLM-short run low gray level emphasis

original-GLSZM-gray level nonuniformity

original-GLSZM-gray level nonuniformity normalized

original-GLSZM-gray level variance

original-GLSZM-high gray level zone emphasis

original-GLSZM-large area emphasis

original-GLSZM-large area high gray level emphasis

original-GLSZM-large area low gray level emphasis

original-GLSZM-low gray level zone emphasis

original-GLSZM-size zone nonuniformity

original-GLSZM-size zone nonuniformity normalized

original-GLSZM-small area emphasis

original-GLSZM-small area high gray level emphasis

original-GLSZM-small area low gray level emphasis

original-GLSZM-zone entropy

original-GLSZM-zone percentage

original-GLSZM-zone variance

log-sigma-1-0-mm-3D-GLSZM-gray level nonuniformity

log-sigma-1-0-mm-3D-GLSZM-gray level nonuniformity normalized

log-sigma-1-0-mm-3D-GLSZM-gray level variance

log-sigma-1-0-mm-3D-GLSZM-high gray level zone emphasis

log-sigma-1-0-mm-3D-GLSZM-large area emphasis

log-sigma-1-0-mm-3D-GLSZM-large area high gray level emphasis

log-sigma-1-0-mm-3D-GLSZM-large area low gray level emphasis

log-sigma-1-0-mm-3D-GLSZM-low gray level zone emphasis

log-sigma-1-0-mm-3D-GLSZM-size zone nonuniformity

log-sigma-1-0-mm-3D-GLSZM-size zone nonuniformity normalized

log-sigma-1-0-mm-3D-GLSZM-small area emphasis

log-sigma-1-0-mm-3D-GLSZM-small area high gray level emphasis

log-sigma-1-0-mm-3D-GLSZM-small area low gray level emphasis

log-sigma-1-0-mm-3D-GLSZM-zone entropy

log-sigma-1-0-mm-3D-GLSZM-zone percentage

log-sigma-1-0-mm-3D-GLSZM-zone variance

log-sigma-2-0-mm-3D-GLSZM-gray level nonuniformity

log-sigma-2-0-mm-3D-GLSZM-gray level nonuniformity normalized

log-sigma-2-0-mm-3D-GLSZM-gray level variance

log-sigma-2-0-mm-3D-GLSZM-high gray level zone emphasis

log-sigma-2-0-mm-3D-GLSZM-large area emphasis

log-sigma-2-0-mm-3D-GLSZM-large area high gray level emphasis

log-sigma-2-0-mm-3D-GLSZM-large area low gray level emphasis

log-sigma-2-0-mm-3D-GLSZM-low gray level zone emphasis

log-sigma-2-0-mm-3D-GLSZM-size zone nonuniformity

log-sigma-2-0-mm-3D-GLSZM-size zone nonuniformity normalized

log-sigma-2-0-mm-3D-GLSZM-small area emphasis

log-sigma-2-0-mm-3D-GLSZM-small area high gray level emphasis

log-sigma-2-0-mm-3D-GLSZM-small area low gray level emphasis

log-sigma-2-0-mm-3D-GLSZM-zone entropy

log-sigma-2-0-mm-3D-GLSZM-zone percentage

log-sigma-2-0-mm-3D-GLSZM-zone variance

log-sigma-3-0-mm-3D-GLSZM-gray level nonuniformity

log-sigma-3-0-mm-3D-GLSZM-gray level nonuniformity normalized

log-sigma-3-0-mm-3D-GLSZM-gray level variance

log-sigma-3-0-mm-3D-GLSZM-high gray level zone emphasis

log-sigma-3-0-mm-3D-GLSZM-large area emphasis

log-sigma-3-0-mm-3D-GLSZM-large area high gray level emphasis

log-sigma-3-0-mm-3D-GLSZM-large area low gray level emphasis

log-sigma-3-0-mm-3D-GLSZM-low gray level zone emphasis

log-sigma-3-0-mm-3D-GLSZM-size zone nonuniformity

log-sigma-3-0-mm-3D-GLSZM-size zone nonuniformity normalized

log-sigma-3-0-mm-3D-GLSZM-small area emphasis

log-sigma-3-0-mm-3D-GLSZM-small area high gray level emphasis

log-sigma-3-0-mm-3D-GLSZM-small area low gray level emphasis

log-sigma-3-0-mm-3D-GLSZM-zone entropy

log-sigma-3-0-mm-3D-GLSZM-zone percentage

log-sigma-3-0-mm-3D-GLSZM-zone variance

log-sigma-4-0-mm-3D-GLSZM-gray level nonuniformity

log-sigma-4-0-mm-3D-GLSZM-gray level nonuniformity normalized

log-sigma-4-0-mm-3D-GLSZM-gray level variance

log-sigma-4-0-mm-3D-GLSZM-high gray level zone emphasis

log-sigma-4-0-mm-3D-GLSZM-large area emphasis

log-sigma-4-0-mm-3D-GLSZM-large area high gray level emphasis

log-sigma-4-0-mm-3D-GLSZM-large area low gray level emphasis

log-sigma-4-0-mm-3D-GLSZM-low gray level zone emphasis

log-sigma-4-0-mm-3D-GLSZM-size zone nonuniformity

log-sigma-4-0-mm-3D-GLSZM-size zone nonuniformity normalized

log-sigma-4-0-mm-3D-GLSZM-small area emphasis

log-sigma-4-0-mm-3D-GLSZM-small area high gray level emphasis

log-sigma-4-0-mm-3D-GLSZM-small area low gray level emphasis

log-sigma-4-0-mm-3D-GLSZM-zone entropy

log-sigma-4-0-mm-3D-GLSZM-zone percentage

log-sigma-4-0-mm-3D-GLSZM-zone variance

log-sigma-5-0-mm-3D-GLSZM-gray level nonuniformity

log-sigma-5-0-mm-3D-GLSZM-gray level nonuniformity normalized

log-sigma-5-0-mm-3D-GLSZM-gray level variance

log-sigma-5-0-mm-3D-GLSZM-high gray level zone emphasis

log-sigma-5-0-mm-3D-GLSZM-large area emphasis

log-sigma-5-0-mm-3D-GLSZM-large area high gray level emphasis

log-sigma-5-0-mm-3D-GLSZM-large area low gray level emphasis

log-sigma-5-0-mm-3D-GLSZM-low gray level zone emphasis

log-sigma-5-0-mm-3D-GLSZM-size zone nonuniformity

log-sigma-5-0-mm-3D-GLSZM-size zone nonuniformity normalized

log-sigma-5-0-mm-3D-GLSZM-small area emphasis

log-sigma-5-0-mm-3D-GLSZM-small area high gray level emphasis

log-sigma-5-0-mm-3D-GLSZM-small area low gray level emphasis

log-sigma-5-0-mm-3D-GLSZM-zone entropy

log-sigma-5-0-mm-3D-GLSZM-zone percentage

log-sigma-5-0-mm-3D-GLSZM-zone variance

wavelet-HHH-GLSZM-gray level nonuniformity

wavelet-HHH-GLSZM-gray level nonuniformity normalized

wavelet-HHH-GLSZM-gray level variance

wavelet-HHH-GLSZM-high gray level zone emphasis

wavelet-HHH-GLSZM-large area emphasis

wavelet-HHH-GLSZM-large area high gray level emphasis

wavelet-HHH-GLSZM-large area low gray level emphasis

wavelet-HHH-GLSZM-low gray level zone emphasis

wavelet-HHH-GLSZM-size zone nonuniformity

wavelet-HHH-GLSZM-size zone nonuniformity normalized

wavelet-HHH-GLSZM-small area emphasis

wavelet-HHH-GLSZM-small area high gray level emphasis

wavelet-HHH-GLSZM-small area low gray level emphasis

wavelet-HHH-GLSZM-zone entropy

wavelet-HHH-GLSZM-zone percentage

wavelet-HHH-GLSZM-zone variance

wavelet-HHL-GLSZM-gray level nonuniformity

wavelet-HHL-GLSZM-gray level nonuniformity normalized

wavelet-HHL-GLSZM-gray level variance

wavelet-HHL-GLSZM-high gray level zone emphasis

wavelet-HHL-GLSZM-large area emphasis

wavelet-HHL-GLSZM-large area high gray level emphasis

wavelet-HHL-GLSZM-large area low gray level emphasis

wavelet-HHL-GLSZM-low gray level zone emphasis

wavelet-HHL-GLSZM-size zone nonuniformity

wavelet-HHL-GLSZM-size zone nonuniformity normalized

wavelet-HHL-GLSZM-small area emphasis

wavelet-HHL-GLSZM-small area high gray level emphasis

wavelet-HHL-GLSZM-small area low gray level emphasis

wavelet-HHL-GLSZM-zone entropy

wavelet-HHL-GLSZM-zone percentage

wavelet-HHL-GLSZM-zone variance

wavelet-HLH-GLSZM-gray level nonuniformity

wavelet-HLH-GLSZM-gray level nonuniformity normalized

wavelet-HLH-GLSZM-gray level variance

wavelet-HLH-GLSZM-high gray level zone emphasis

wavelet-HLH-GLSZM-large area emphasis

wavelet-HLH-GLSZM-large area high gray level emphasis

wavelet-HLH-GLSZM-large area low gray level emphasis

wavelet-HLH-GLSZM-low gray level zone emphasis

wavelet-HLH-GLSZM-size zone nonuniformity

wavelet-HLH-GLSZM-size zone nonuniformity normalized

wavelet-HLH-GLSZM-small area emphasis

wavelet-HLH-GLSZM-small area high gray level emphasis

wavelet-HLH-GLSZM-small area low gray level emphasis

wavelet-HLH-GLSZM-zone entropy

wavelet-HLH-GLSZM-zone percentage

wavelet-HLH-GLSZM-zone variance

wavelet-HLL-GLSZM-gray level nonuniformity

wavelet-HLL-GLSZM-gray level nonuniformity normalized

wavelet-HLL-GLSZM-gray level variance

wavelet-HLL-GLSZM-high gray level zone emphasis

wavelet-HLL-GLSZM-large area emphasis

wavelet-HLL-GLSZM-large area high gray level emphasis

wavelet-HLL-GLSZM-large area low gray level emphasis

wavelet-HLL-GLSZM-low gray level zone emphasis

wavelet-HLL-GLSZM-size zone nonuniformity

wavelet-HLL-GLSZM-size zone nonuniformity normalized

wavelet-HLL-GLSZM-small area emphasis

wavelet-HLL-GLSZM-small area high gray level emphasis

wavelet-HLL-GLSZM-small area low gray level emphasis

wavelet-HLL-GLSZM-zone entropy

wavelet-HLL-GLSZM-zone percentage

wavelet-HLL-GLSZM-zone variance

wavelet-LHH-GLSZM-gray level nonuniformity

wavelet-LHH-GLSZM-gray level nonuniformity normalized

wavelet-LHH-GLSZM-gray level variance

wavelet-LHH-GLSZM-high gray level zone emphasis

wavelet-LHH-GLSZM-large area emphasis

wavelet-LHH-GLSZM-large area high gray level emphasis

wavelet-LHH-GLSZM-large area low gray level emphasis

wavelet-LHH-GLSZM-low gray level zone emphasis

wavelet-LHH-GLSZM-size zone nonuniformity

wavelet-LHH-GLSZM-size zone nonuniformity normalized

wavelet-LHH-GLSZM-small area emphasis

wavelet-LHH-GLSZM-small area high gray level emphasis

wavelet-LHH-GLSZM-small area low gray level emphasis

wavelet-LHH-GLSZM-zone entropy

wavelet-LHH-GLSZM-zone percentage

wavelet-LHH-GLSZM-zone variance

wavelet-LHL-GLSZM-gray level nonuniformity

wavelet-LHL-GLSZM-gray level nonuniformity normalized

wavelet-LHL-GLSZM-gray level variance

wavelet-LHL-GLSZM-high gray level zone emphasis

wavelet-LHL-GLSZM-large area emphasis

wavelet-LHL-GLSZM-large area high gray level emphasis

wavelet-LHL-GLSZM-large area low gray level emphasis

wavelet-LHL-GLSZM-low gray level zone emphasis

wavelet-LHL-GLSZM-size zone nonuniformity

wavelet-LHL-GLSZM-size zone nonuniformity normalized

wavelet-LHL-GLSZM-small area emphasis

wavelet-LHL-GLSZM-small area high gray level emphasis

wavelet-LHL-GLSZM-small area low gray level emphasis

wavelet-LHL-GLSZM-zone entropy

wavelet-LHL-GLSZM-zone percentage

wavelet-LHL-GLSZM-zone variance

wavelet-LLH-GLSZM-gray level nonuniformity

wavelet-LLH-GLSZM-gray level nonuniformity normalized

wavelet-LLH-GLSZM-gray level variance

wavelet-LLH-GLSZM-high gray level zone emphasis

wavelet-LLH-GLSZM-large area emphasis

wavelet-LLH-GLSZM-large area high gray level emphasis

wavelet-LLH-GLSZM-large area low gray level emphasis

wavelet-LLH-GLSZM-low gray level zone emphasis

wavelet-LLH-GLSZM-size zone nonuniformity

wavelet-LLH-GLSZM-size zone nonuniformity normalized

wavelet-LLH-GLSZM-small area emphasis

wavelet-LLH-GLSZM-small area high gray level emphasis

wavelet-LLH-GLSZM-small area low gray level emphasis

wavelet-LLH-GLSZM-zone entropy

wavelet-LLH-GLSZM-zone percentage

wavelet-LLH-GLSZM-zone variance

wavelet-LLL-GLSZM-gray level nonuniformity

wavelet-LLL-GLSZM-gray level nonuniformity normalized

wavelet-LLL-GLSZM-gray level variance

wavelet-LLL-GLSZM-high gray level zone emphasis

wavelet-LLL-GLSZM-large area emphasis

wavelet-LLL-GLSZM-large area high gray level emphasis

wavelet-LLL-GLSZM-large area low gray level emphasis

wavelet-LLL-GLSZM-low gray level zone emphasis

wavelet-LLL-GLSZM-size zone nonuniformity

wavelet-LLL-GLSZM-size zone nonuniformity normalized

wavelet-LLL-GLSZM-small area emphasis

wavelet-LLL-GLSZM-small area high gray level emphasis

wavelet-LLL-GLSZM-small area low gray level emphasis

wavelet-LLL-GLSZM-zone entropy

wavelet-LLL-GLSZM-zone percentage

wavelet-LLL-GLSZM-zone variance
